# A Cluster-Randomized Trial of Two Strategies to Improve Antibiotic Use for Patients with a Complicated Urinary Tract Infection

**DOI:** 10.1371/journal.pone.0142672

**Published:** 2015-12-04

**Authors:** Veroniek Spoorenberg, Marlies E. J. L. Hulscher, Ronald B. Geskus, Theo M. de Reijke, Brent C. Opmeer, Jan M. Prins, Suzanne E. Geerlings

**Affiliations:** 1 Department of Internal Medicine, Division of Infectious Diseases, Centre for Infection and Immunity Amsterdam, Academic Medical Centre, Amsterdam, The Netherlands; 2 Scientific Institute for Quality of Healthcare, Radboud University Nijmegen Medical Centre, Nijmegen, The Netherlands; 3 Department of Clinical Epidemiology, Biostatistics and Bioinformatics, Academic Medical Centre, Amsterdam, The Netherlands; 4 Department of Urology, Academic Medical Centre, Amsterdam, The Netherlands; 5 Clinical Research Unit, Academic Medical Centre, Amsterdam, The Netherlands; Glaxo Smith Kline, DENMARK

## Abstract

**Background:**

Up to 50% of hospital antibiotic use is inappropriate and therefore improvement strategies are urgently needed. We compared the effectiveness of two strategies to improve the quality of antibiotic use in patients with a complicated urinary tract infection (UTI).

**Methods:**

In a multicentre, cluster-randomized trial 19 Dutch hospitals (departments Internal Medicine and Urology) were allocated to either a multi-faceted strategy including feedback, educational sessions, reminders and additional/optional improvement actions, or a competitive feedback strategy, i.e. providing professionals with non-anonymous comparative feedback on the department’s appropriateness of antibiotic use. Retrospective baseline- and post-intervention measurements were performed in 2009 and 2012 in 50 patients per department, resulting in 1,964 and 2,027 patients respectively. Principal outcome measures were nine validated guideline-based quality indicators (QIs) that define appropriate antibiotic use in patients with a complicated UTI, and a QI sumscore that summarizes for each patient the appropriateness of antibiotic use.

**Results:**

Performance scores on several individual QIs showed improvement from baseline to post-intervention measurements, but no significant differences were found between both strategies. The mean patient’s QI sum score improved significantly in both strategy groups (multi-faceted: 61.7% to 65.0%, P = 0.04 and competitive feedback: 62.8% to 66.7%, P = 0.01). Compliance with the strategies was suboptimal, but better compliance was associated with more improvement.

**Conclusion:**

The effectiveness of both strategies was comparable and better compliance with the strategies was associated with more improvement. To increase effectiveness, improvement activities should be rigorously applied, preferably by a locally initiated multidisciplinary team.

**Trial Registration:**

Nederlands Trial Register 1742

## Introduction

Complicated Urinary Tract Infections (UTIs) are among the most prevalent infectious diseases [1,2], substantially contributing to antibiotic use in the hospital setting. According to medical literature, up to 50% of hospital antibiotic use is inappropriate [3–5]. Inappropriate antibiotic use has been associated with an increase in morbidity, mortality, length of stay, hospital costs and raising bacterial resistance [6–10]. In a previous study, we defined appropriate antibiotic use for patients with a complicated UTI with a valid set of nine guideline-based quality indicators (QIs) (Table 1) and showed in a pilot study large room for improvement on most QIs [11].

**Table 1:** Set of Quality Indicators [11].

	Quality indicators
1	Perform a urine culture
2	Prescribe empirical therapy in accordance with the national guideline
3	Switch from intravenous to oral therapy within 72 h on the basis of the clinical condition
4	Tailor antibiotic treatment on the basis of culture results
5	Use fluoroquinolones selectively (oral therapy, or in case of anaphylaxis to beta-lactam antibiotics)
6	Duration of antibiotic therapy should be at least 10 days (in accordance with the national guideline)
7*	Treat UTI in men in accordance with the national guideline
8	Replace urinary catheter after initiation of antibiotic treatment
9	Adapt antibiotic dose according to renal function

* Additional QI for men with a UTI. This QI applies to (denominator) men with a UTI, including those with a chronic prostatitis. It evaluates (numerator) whether they were treated in accordance with the guideline regarding empirical therapy and treatment duration and, in case of chronic prostatitis, whether they were treated with culture-guided therapy for the recommended duration.

To improve appropriate antibiotic use, Antimicrobial Stewardship Programs (ASPs) are propagated [12], which can be considered as ‘a menu of interventions that can be designed and adapted to fit the infrastructure of any hospital’ [13]. This menu suggests various improvement interventions of which it has been shown that they effectively improve antibiotic prescribing in hospitals [3]. Unfortunately, direct comparisons of the effectiveness of these different improvement interventions in a methodological powerful design are scarce [3–14]. Such head-to-head comparisons, for which a cluster-randomized trial design is considered to be the ideal [3], are urgently needed to extend the current evidence for effectively improving antibiotic prescribing [3,15].

Therefore, we conducted a cluster-randomized trial of two interventions, or strategies, to improve antibiotic use in patients with a complicated UTI. We aimed to assess the effectiveness, measured as the before-and-after-intervention performance on the QIs, of two improvement strategies: 1) a Multi-Faceted Strategy, comparable to an effective strategy to improve antibiotic use in patients with lower respiratory tract infections [16] and 2) a ‘Competitive Feedback Strategy’, i.e. providing professionals with non-anonymous comparative feedback on the department’s appropriateness of antibiotic use in patients with complicated UTIs. Additionally, we aimed to identify determinants of successful improvement.

## Methods

### Design and setting

We conducted a multicentre, cluster-randomized trial to compare the effectiveness of two different strategies to improve the appropriateness of antibiotic use in patients with a complicated UTI (*QUality of ANtibiotic use in uTI patients* (QUANTI) trial, http://www.trialregister.nl; NTR 1742).

**Fig 1:**
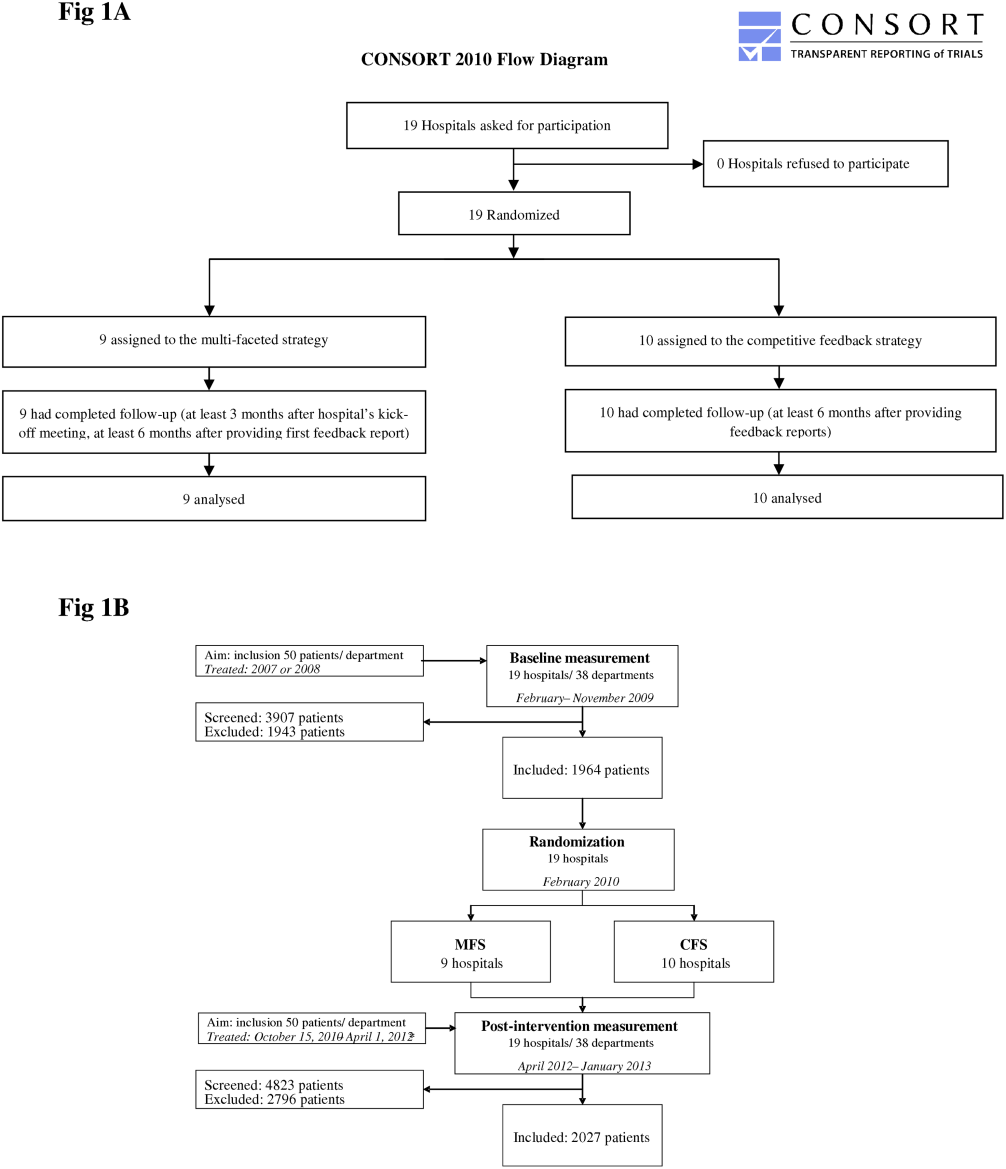
Flow charts of study design and participants; MFS = Multi-Faceted Strategy, CFS = Competitive Feedback Strategy. * CFS: patients enrolled from October 15, 2010: enrolment in post-intervention measurement at least 6 months after providing the feedback reports. MFS: patient enrolment in post-intervention measurement depended on the individual implementation schedule: at least 3 months after the hospital’s kick-off meeting, and at least 6 months after providing the first feedback report.

Between February and November 2009 a retrospective baseline measurement was performed at the Internal Medicine and Urology departments of 19 university and non-university hospitals located throughout the Netherlands. By February 2010, hospitals were randomly allocated to one of the improvement strategies. Between April 15 and October 15, 2010, in each hospital the allocated improvement strategy was implemented. From six months later patients were included in a post-intervention measurement (exact starting point differed by hospital, see Fig 1b). For both the baseline and post-intervention measurement the minimal sample size was 50 patients per department. Enrolment of this number of patients took on average, depending on the department, 1.5 years, so (retrospective) post-intervention measurement could not start before April 2012.

The medical ethical committee of the Academic Medical Centre Amsterdam considered our study and concluded that it was deemed exempt from their approval (ref 08.17.1775). No informed consent was obtained from patients because no interventions at the patient level were done and patient data were analysed in a retrospective design anonymously, for the aim to improve quality or healthcare.

Informed consent was obtained from the contact persons of the participating hospitals.

### Patient selection

From the hospital diagnosis registration system patients were screened for nationally defined categories: cystitis, pyelonephritis, prostatitis and bacteremia. Included were adults (≥ 16 years) who were referred to the hospital (inpatient/outpatient clinic) and diagnosed by an internist or urologist with a complicated UTI (including catheter-associated UTIs) as main diagnosis and treated as such. We defined a complicated UTI as a UTI with one of the following characteristics: male gender, pregnancy, any functional or anatomical abnormality of the urinary tract, immunocompromising disease or medication, or a UTI with symptoms of tissue invasion or systemic infection (pyelonephritis, urosepsis, prostatitis) [17].

### Multi-faceted strategy (MFS)

This strategy was comparable to an improvement strategy developed by Schouten et al. [16] that effectively improved antibiotic use in patients with lower respiratory tract infections in a cluster-randomized trial in six Dutch hospitals. Their strategy contained standardized elements that were used in all hospitals and additional interventions that were locally adjusted to the needs and wishes of that specific hospital. The antibiotic use QIs that were most in need of improvement were given priority. All interventions were initiated and coordinated by the researcher and a project quality improvement officer.

**Fig 2:**
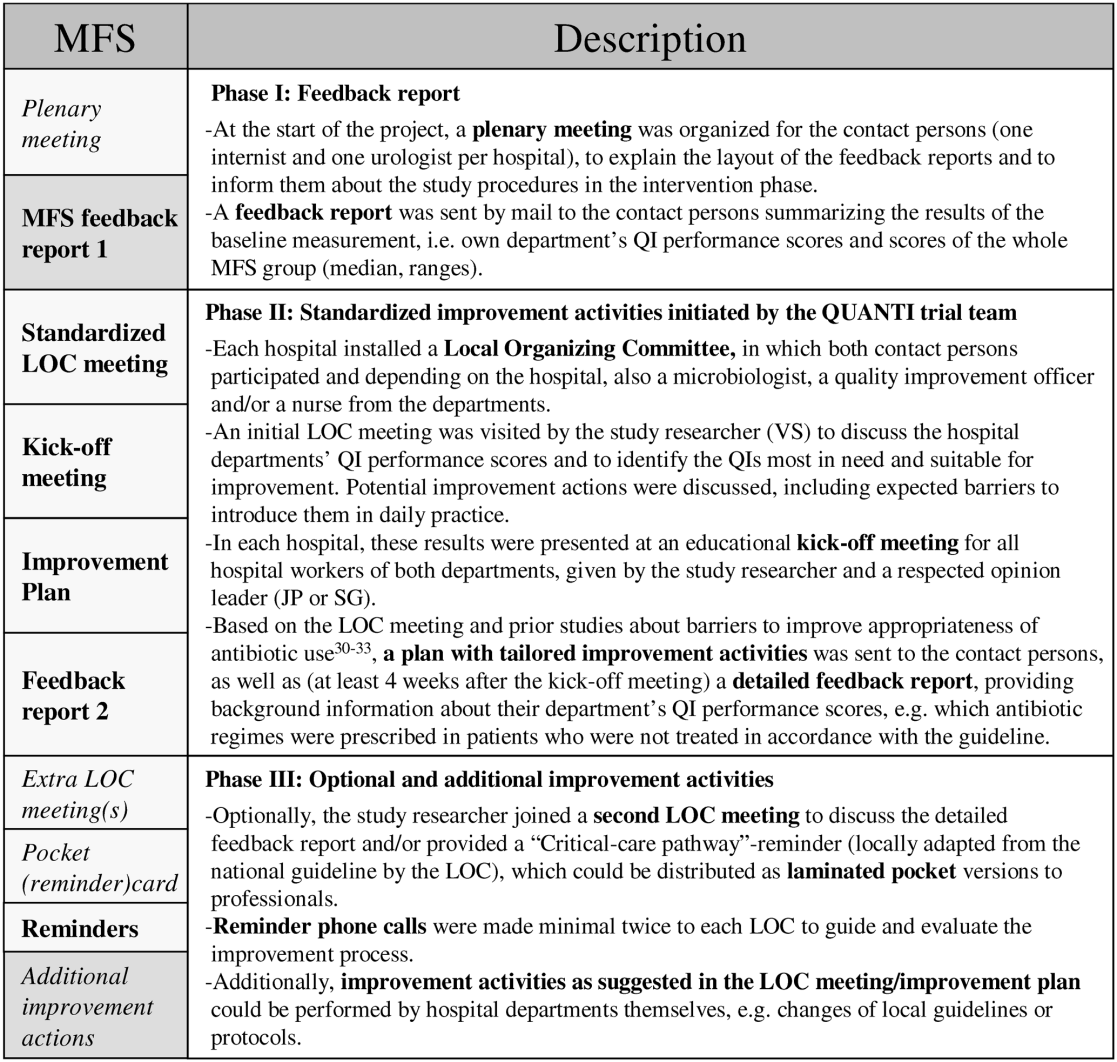
Description of standardized (bold) and optional (italic) elements of the multi-faceted strategy (MFS) [30–33].

**Fig 3:**
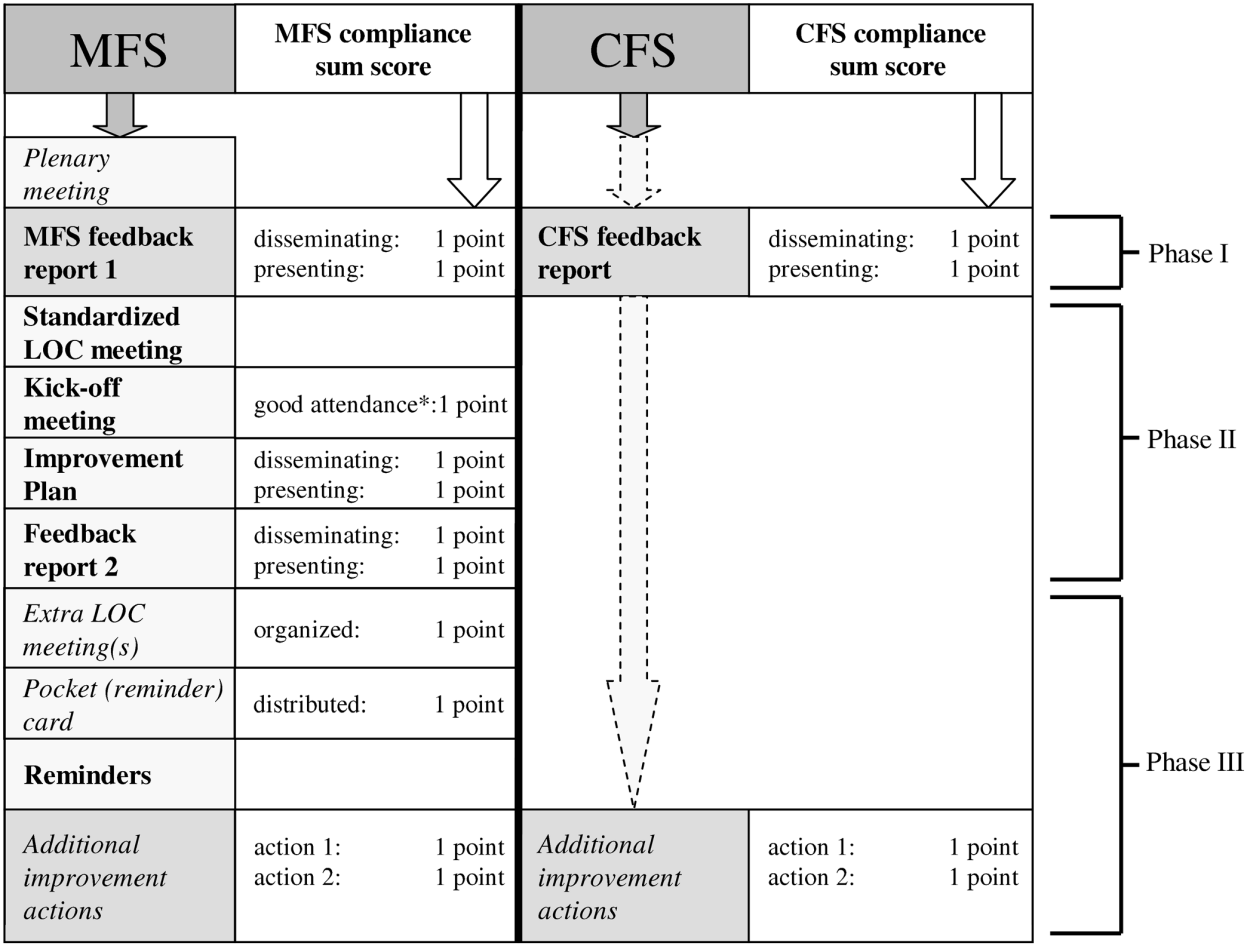
Activities and compliance scores per strategy; MFS = Multi-Faceted Strategy, CFS = Competitive Feedback Strategy. Elements in bold are standardized strategy elements; elements in italics are optional. LOC: Local Organizing Committee. * more than the median score of all departments.

Our MFS consisted of comparable standardized elements, but more strongly involved local professionals in the design and performance of the locally tailored interventions. The intervention consisted of three separate phases (see Figs 2 and 3).

### Competitive feedback strategy (CFS)

In this era of transparency, it is becoming increasingly common to release performance data–either individually or publicly–with the aim of improving healthcare performance [18]. Individual audit and feedback is widely used to improve practice by giving professionals insight into their own performance [15]. Simultaneously, performance data are released into the public domain (i.e. public reporting), aiming to improve quality by ranking performance of different providers [19–21]. We combined the best of these two approaches, by providing individual feedback to the professionals by non-anonymously ranking the various departments. In this manner we aimed to include the competitive element of public reporting in the individual feedback strategy, resulting in a so-called competitive feedback strategy.

Competitive feedback reports contained, for each QI, a list of all 38 departments’ performance scores, in which the names of the MFS departments were blinded, but the others were visible. For reasons of confidentiality, feedback reports were sent by regular mail and receipt of the reports was verified.

### Variables and data collection

#### Effect parameters: Quality indicators for appropriate antibiotic use

The appropriateness of antibiotic use was scored using QIs which are based on the treatment recommendations from the Dutch national, evidence-based guideline for the antimicrobial treatment of complicated UTIs [22]. In an earlier study, a 3-step modified RAND Delphi approach among experts was used to systematically develop a set of nine QIs, which was subsequently validated [11] (Table 1). All QIs are dichotomous variables, distinguishing appropriate from inappropriate antibiotic use in each individual patient. Data were collected by retrospective chart review by the study researcher together with one (baseline) or two (post-intervention) trained research assistants. The assistants were blinded for the strategy status of the hospitals. QI performance was calculated for each patient using previously constructed algorithms [10].

To summarize patients’ performance on the different QIs in one outcome measure, we calculated − at baseline- and post-intervention measurement − for each patient a total QI set performance, defined as the patient’s QI sumscore divided by the number of QIs that applied to that specific patient [10].

#### Determinants of improvement

We aimed to identify determinants of successful improvement, because insight into the mechanisms responsible for the results could enhance the validity of the findings and might help to understand the potential generalizability of the strategies [23–24]. We examined whether the following variables were associated with improved total QI set performance: compliance with the strategies, department’s baseline performance on the total QI set, inpatient versus outpatient and Internal Medicine versus Urology.

***Compliance with improvement strategies***: To measure compliance with the various elements of the improvement strategies, we assessed for all departments which improvement activities were actually performed, e.g. whether feedback reports and the improvement plan were disseminated and/or presented by the local contact person and whether/what additional improvement actions were carried out. Compliance data were collected using a questionnaire filled in by the contact persons (February 2011). The attendance of local professionals at the kick-off meetings was registered by the study researcher. A sum score was calculated reflecting the compliance per department (Fig 3).

***Department’s baseline performance on the total QI set***: For each department, a baseline performance score was assessed by calculating the mean total QI set performance of all patients of that department during the baseline measurement.

### Sample size

We anticipated a difference between the two strategies in QI adherence of 15% in favour of the MFS after the intervention (70% versus 55%). Intraclass correlations (ICC) calculated in our QI development study [11] for three of our QIs showed a mean ICC of 0.10. Using alpha = 0.05, two-sided testing, power = 0.80 and ICC = 0.10, we needed 18 clusters of 250 individuals per strategy if only one indicator was measured per individual. Since we measured more indicators per individual, and assuming a correlation of 0.5 between the indicator values from the same individual, the number of individuals per cluster could be reduced by a factor five, requiring a sample size of 18 clusters of 50 patients each per strategy. The total number of patients that should be included for the trial was 3600 patients (18 (clusters) * 50 (patients) * 2 (strategies) * 2 (baseline- and post-intervention measurement)).

### Statistical analysis

Randomization of the hospitals was balanced by minimization on hospital’s baseline individual QI performance scores and was performed by a statistician who was blinded to the composition of the groups.

As descriptive statistics, we give the percentage appropriate antibiotic use per QI, and means for total QI set performance. Not every QI was applicable to all included patients, therefore the sample sizes of the QIs varied. For a QI to be included in the analyses, we decided that the minimum sample size was a mean of 15 patients per department in the baseline measurement.

The effectiveness of the strategies on the individual QIs was assessed by multilevel logistic regression analysis, with clusters determined by the unique hospital-department combinations. We allowed the improvement from baseline to post-intervention measurement to differ by strategy. In an adjusted analysis we controlled for the patient characteristics gender, age, urological comorbidity, diabetes and being in- or outpatient.

To test the effectiveness of the strategies on the total QI set performance, multilevel linear regression analysis was performed, in which the residual variance was weighted for the number of QIs that applied to the individual patient. Hence, we assumed a normal distribution of the total QI set performance. Although this is not correct for the individual total QI values, its disturbing effect on the parameter estimates may be reduced due to the large sample size. As an alternative, we performed a logistic regression analysis in which all individual QIs were included. In this model, we assumed that the average value was equal for every QI and that there was no correlation between QI performance scores within a patient.

We also used multilevel linear regression analysis to test for each strategy the association between possible determinants of successful improvement (compliance with strategies, department’s baseline total QI set performance, inpatient/outpatient, Internal Medicine/Urology) and the total QI set performance. For the effect of baseline performance, we regressed the individual post-intervention total QI set performance on the mean value per department at baseline. We performed the analyses using R version 3.0.1 and the lme4 package [25,26]. A value of P < 0.05 was considered statistically significant.

## Results

### Study population

The baseline population consisted of 1,964 patients, the post-intervention population of 2,027 patients (Fig 1b). Their characteristics are described in Table 2. Of the 19 included hospitals 4 were university hospitals, of which 3 were randomized to the CFS group and 1 to the MFS group.

**Table 2:** Patient characteristics at baseline and post-intervention; MFS = Multi-Faceted Strategy, CFS = Competitive Feedback Strategy.

	Baseline population (T0)		Post-intervention population (T1)	
	MFS (n = 923)	CFS (n = 1041)	MFS (n = 963)	CFS (n = 1064)
Men, n (%)	538 (58.3)	596 (57.3)	562 (58.4)	651 (61.2)
Age, years (mean; SD)	61.7 (20.1)	61.4 (20.0)	62.9 (19.4)	63.7 (17.7)
Urological comorbidity, n (%)	179 (19.4)	227 (21.8)	206 (21.4)	204 (19.2)
Diabetes, n (%)	196 (21.2)	185 (17.8)	230 (23.9)	205 (19.3)
Urinary catheter, n (%)	129 (14.0)	143 (13.7)	149 (15.5)	156 (14.7)
Outpatient, n (%)	337 (36.5)	313 (30.1)	357 (37.1)	404 (38.0)
Internal Medicine, n (%)	460 (49.8)	521 (50.0)	484 (50.3)	541 (50.8)

For all characteristics: missing data in < 3 patients.

### Effectiveness of strategies

Table 3 shows for each strategy the baseline (T0) and post-intervention (T1) performance on the individual QIs and total QI set performance. Three QIs did not reach the minimum sample size to be included in the analyses, because they were not applicable in enough patients: ‘Use fluoroquinolones selectively’, ‘Replace catheter after initiation of treatment’, and ‘Adapt antibiotic dose according to renal function’.

**Table 3:** Performance on quality indicators before (T0) and after intervention (T1).

	Quality indicator	Multi-faceted strategy ^a^			Competitive feedback strategy ^a^			Odds ratio (95% CI) ^b^	P ^b^
		T0	T1	T1-T0	T0	T1	T1-T0		
		n = 923	n = 963	P ^b^	n = 1041	n = 1064	P ^b^		
1	Perform a urine culture, n	669/922	769/961		800/1040	888/1064			
	%	72.6	80.0	**+ 7.4**	76.9	83.5	**+ 6.6**	0.99	0.98
	P			**0.01**			**0.008**	(0.64;1.55)	
2	Prescribe according to national guideline, n	447/679	450/670		531/786	498/703			
	%	65.8	67.2	+ 1.4	67.6	70.8	+ 3.2	0.89	0.59
	P			0.83			0.32	(0.59;1.35)	
3	Switch from i.v. to oral therapy within 72 hours, n	127/243	124/251		177/317	170/282			
	%	52.3	49.4	- 2.9	55.8	60.3	+ 4.5	0.68	0.31
	P			0.47			0.48	(0.32;1.44)	
4	Tailor antibiotic treatment based on culture result, n	390/513	438/549		480/648	535/661			
	%	76.0	79.8	+ 3.8	74.1	80.9	**+ 6.8**	0.81	0.36
	P			0.46			**0.03**	(0.51;1.28)	
6	Treatment duration should be at least 10 days, n	396/720	418/761		444/833	469/882			
	%	55.0	54.9	- 0.1	53.3	53.2	- 0.1	1.04	0.86
	P			0.78			0.97	(0.71;1.51)	
7	Treat UTI in men according to national guideline, n	156/434	172/441		160/482	202/527			
	%	35.9	39.0	+ 3.1	33.2	38.3	**+ 5.1**	0.90	0.62
	P			0.23			**0.047**	(0.61;1.34)	
	Total QI set performance (%) ^c^; mean of all patients	61.7	65.0	**+3.3**	62.8	66.7	**+ 3.9**		0.77
	P			**0.043**			**0.010**		

^a^ Missing data in < 10 patients; numbers in bold indicate a significant change in QI performance.

^b^ Odds ratios (ORs) for difference between strategies and P-values were adjusted in a multilevel analysis for clustering of patients in departments and hospitals. Data are not adjusted for baseline characteristics.

^c^ Proportion of appropriately performed QIs per patient (QI sumscore divided by number of applicable QIs).

For the total sample, several performance scores on the individual QIs showed improvement from baseline to post-intervention measurements. Performing a urine culture increased in both strategy groups (MFS: 72.6% to 80%, P = 0.01 and CFS: 76.9% to 83.5%, P = 0.008). Tailoring antibiotic treatment on the basis of culture results (74.1% to 80.9%, P = 0.03) and treating UTI in men in accordance with the national guideline (33.2% to 38.3%, P = 0.05) improved significantly in the CFS group. No significant differences were found between both strategies in the improvement of individual QI scores (Table 3). Correcting for gender, age, urological comorbidity, diabetes and inpatient/outpatient did not significantly change these results.

The total QI set performance improved significantly in both strategy groups (Table 3), but the difference between the strategies was not statistically significant, neither in the multilevel linear regression analysis nor in the logistic analysis (see Methods).

### Determinants of improvement

#### Compliance with improvement strategies

**Fig 4:**
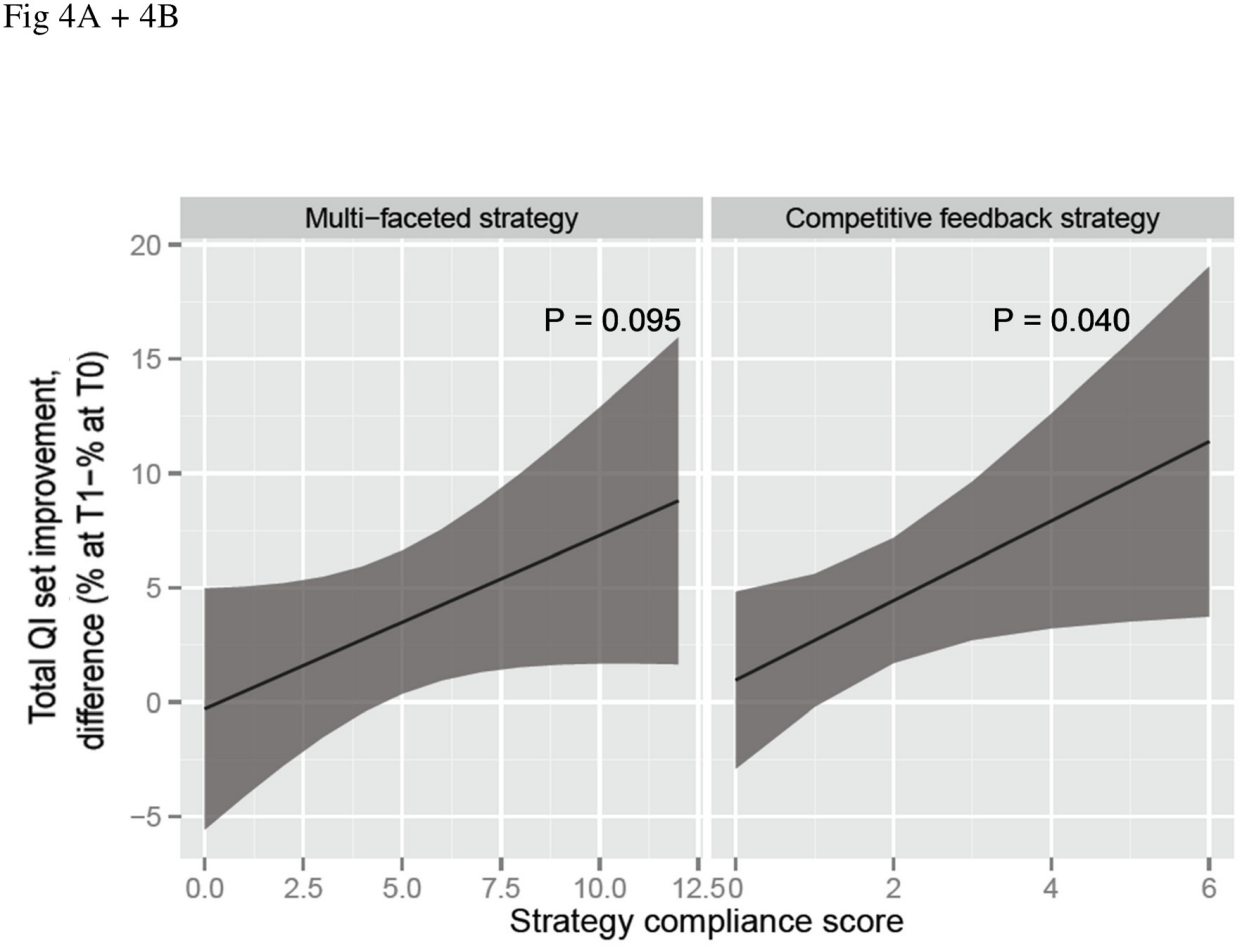
Improvement of the total QI set performance in relation to department’s compliance with improvement strategies. Difference in performance between T1 and T0, 95% CIs are shaded.

In the MFS group, the first feedback report was disseminated and presented in 33% (6/18) of departments, it was either disseminated or presented in 44% (8/18) and it was neither disseminated nor presented in 22% (4/18). For the improvement plan, dissemination and presentation occurred in 28% (5/18), dissemination or presentation in 44% (8/18) and none of them in 28% (5/18). For the second feedback report these percentages were 11%, 27% and 62%, respectively. Additional LOC meetings were organized in 28% (5/18) and pocket cards were distributed in 56% (10/18) of departments. Additional improvement actions were performed in 39% (7/18) of departments (S1 Table). The maximum compliance sum score (Fig 3) was 12, with a median of 4. Fig 4a demonstrates a non-significant association between compliance to the MFS and the total QI set improvement (P = 0.095).

In the CFS group, the first feedback report was disseminated and presented in 30% (6/20) of departments, it was disseminated or presented in 40% (8/20) and in 30% (6/20) it was neither disseminated nor presented. Additional improvement actions were performed in 40% (8/20) of departments (S1 Table). The maximum compliance sum score (Fig 3) was 6, with a median of 1. Fig 4b shows that better compliance to the CFS was significantly associated with total QI set improvement (P = 0.04).

#### Department’s baseline performance on the total QI set

**Fig 5:**
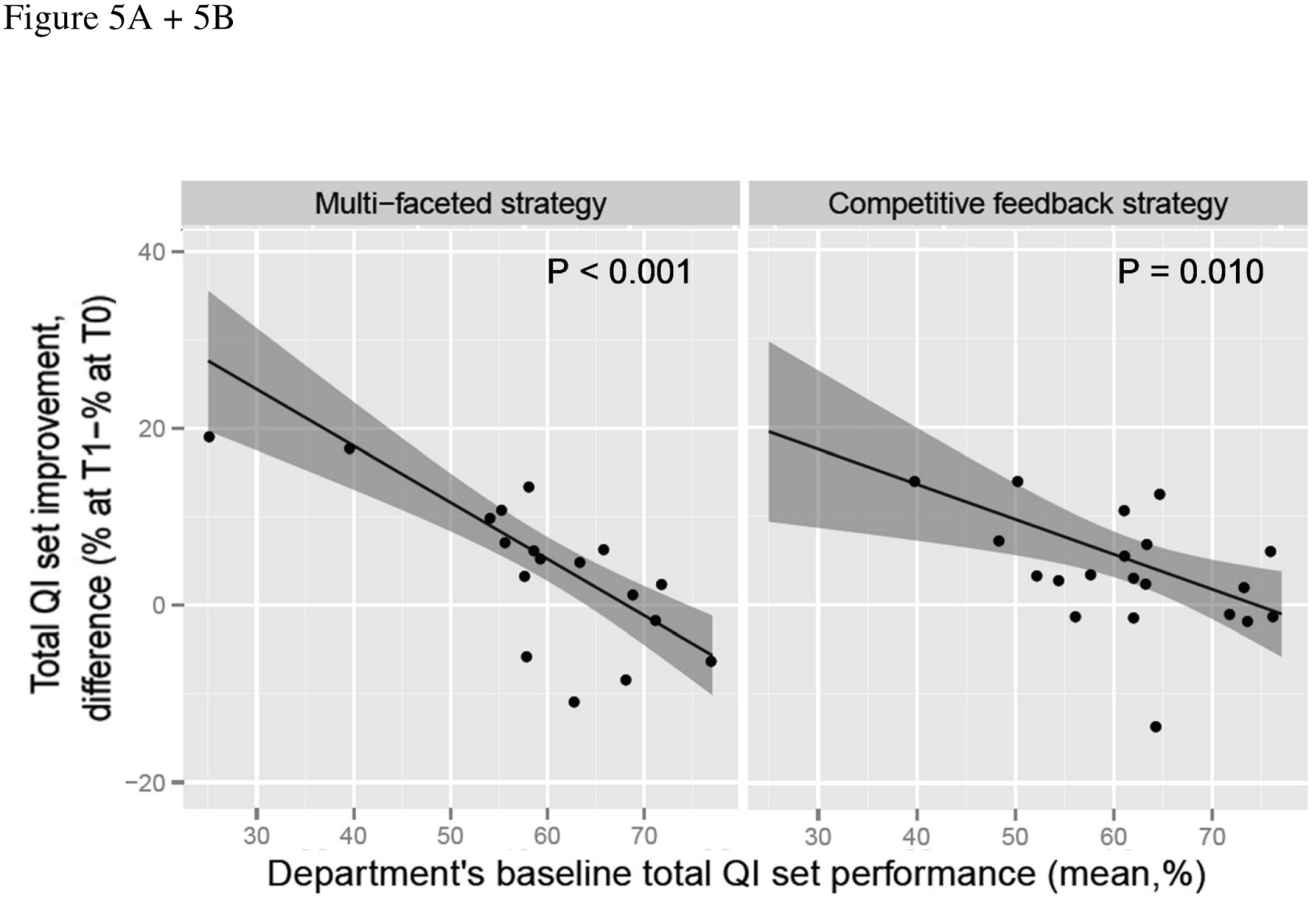
Improvement of the total QI set performance in relation to department’s mean baseline performance on the total QI set. Difference in performance between T1 and T0, 95% CIs are shaded. Dots indicate individual department’s score.

For both strategies, a lower department's mean baseline performance on the total QI set was associated with more total QI set improvement. For both strategies, total QI set performance significantly improved at departments with a mean baseline total QI set performance ≤60% (Fig 5a+b).

#### Inpatients versus outpatients

Improvement on the total QI set performance was comparable for inpatients and outpatients in both strategies (MFS: P = 0.81, CFS: P = 0.21) (Table 4).

**Table 4:** Possible determinants of successful improvement.

Determinants	MFS, Total QI set performance^a^ ^,^ ^b^ %; mean of all patients			P ^c^	CFS, Total QI set performance^a^ ^,^ ^b^ %; mean of all patients			P ^c^
	T0 (n = 923)	T1 (n = 963)	T1-T0, change (95%CI)		T0 (n = 1041)	T1 (n = 1064)	T1-T0, change (95%CI)	
**Inpatient/ outpatient**								
Inpatient	65.5 *(n = 586)*	69.1 *(n = 605)*	+3.6 (-0.29;7.37)	0.81	65.4 *(n = 728)*	71.3 *(n = 660)*	+5.9 (2.70;9.13)	0.21
Outpatient	54.2 *(n = 337)*	57.0 *(n = 357)*	+2.8 (-2.34;7.99)		55.9 *(n = 313)*	58.4 *(n = 404)*	+2.5 (-2.11;7.20)	
**Department**								
Internal Medicine	65.5 *(n = 460)*	67.2 *(n = 484)*	+1.7 (-2.79;6.28)	0.34	67.2 *(n = 521)*	72.8 *(n = 541)*	+5.6 (1.66;9.55)	0.21
Urology	57.9 *(n = 463)*	62.8 *(n = 479)*	+4.9 (0.20;9.70)		58.4 *(n = 520)*	60.4 *(n = 523)*	+2.0 (-2.12;6.11)	

^a^ Missing data in 1 patient (being inpatient or outpatient).

^b^ Proportion of appropriately performed QIs per patient (QI sumscore divided by number of applicable QIs).

^c^ P-values for difference in trend between both subgroup.

#### Internal medicine versus urology

In both strategies, improvement on the total QI set performance did not differ significantly between patients treated at Internal Medicine or Urology departments (MFS: P = 0.34, CFS: P = 0.21) (Table 4).

## Discussion

In this study, we compared the effectiveness of two strategies to improve antibiotic use in patients with a complicated UTI. We found that performance scores on several individual QIs showed improvement from baseline to post-intervention measurements, but no systematic differences between both strategies were found. The total QI set performance improved statistically significantly in both strategy groups. Better compliance with the strategies was associated with more improvement on the total set of QIs. Low department’s baseline performance on the total set of QIs was associated with a larger effect of both improvement strategies.

In our study, the multi-faceted strategy was less effective than the original strategy by Schouten et al., which was developed to improve antibiotic use in patients with lower respiratory tract infections [16]. A possible explanation for this difference might be that in our strategy, after effectuating the standardized intervention elements, performance of the optional and additional improvement activities strongly depended on local professionals, whereas in the original strategy the study researcher and project quality improvement officer actively initiated and coordinated these flexible activities. We considered involvement of local professionals to be a crucial element of our strategy, as this showed to be successful in achieving sustainable improvement [3,27] and in general contributes to the local performance of improvement strategies. However, we probably overestimated the persuasive power of our strategy to involve local professionals. In this light, evaluation of departments’ compliance with the strategy was relevant [24], as it confirmed suboptimal compliance at many departments, and a trend towards more improvement with better compliance. Our results suggest that for improvement strategies initiated from outside the hospital it is difficult to actually engage professionals from the ‘inside’, resulting in less effectiveness of the strategy. This emphasizes the need for a locally initiated multidisciplinary team, as recommended in the development of antimicrobial stewardship programs [12].

Concerning the competitive feedback strategy, better compliance with the strategy was significantly related to more improvement. This relation was stronger than for the MFS. Our compliance evaluation showed that feedback reports were disseminated and/or presented often and additional improvement actions were performed in 40% of departments. This is in contrast with literature suggesting that physicians are sceptical about public data and consider them of minimal use [21,28,29]. In addition, there is no consistent evidence that the public release of performance data improves care [20]. Possibly, our CFS found the appropriate balance between being a stimulus by creating accountability [20] and ensuring enough confidentiality.

Besides compliance with the various elements of the improvement strategies, other variables turned out to be determinants of successful improvement. A low department’s baseline total QI set performance seemed to be associated with a larger effect of both improvement strategies, which is in line with previous studies on improving professional practice [15,23]. However, regression to the mean effects may be partially responsible for these trends.

Furthermore, subgroup analyses showed that for inpatients and outpatients and for the different departments (Internal Medicine or Urology) the size of improvement was comparable.

The major strength of our study is the large study sample including hospitals from all over the Netherlands and the large patient populations, as required by a cluster-randomized trial with different intervention arms. Second, the design of the study and the collection of data regarding multiple aspects of care that were derived from the medical records of each individual patient contribute to the validity of our results. Finally, we not only assessed effectiveness of two strategies, but also provided insight into what variables determine effectiveness. Future improvement studies can build on this insight.

Our study has a few limitations. First, no control group was included, because the major goal was to compare two improvement strategies. Consequently, differences between baseline to post-intervention measurements might be (partially) explained by the Hawthorne effect, where behaviour changes as a result of being under study. However, two recently updated Cochrane reviews state that head-to-head intervention trials are urgently needed [3,15]. Second, we summarized compliance in a sum score, adding all activities together, without taking into account the more detailed content of them. Nevertheless, we demonstrated a positive relationship between better compliance scores and total QI set performance improvement. We earlier demonstrated that better performance on the total set of QIs as such was associated with a shorter length of hospital stay [10].

In conclusion, our results are relevant because they reflect the difficulties in daily practice when introducing–nowadays popular- public reporting strategies or multi-faceted stewardship programs. We think that these improvement strategies and programs should be developed and carried out by a locally initiated multidisciplinary team to improve effectiveness. Equally important, our study shows that the effectiveness of an intervention strategy increases when it is more rigorously applied: a comprehensive set of interventions ultimately results in more improvement. Finally, we introduced a competitive feedback strategy that appeared to be less time-consuming and equally effective in improving QI performance scores, compared to a multi-faceted strategy. This competitive feedback strategy should be tested in a broader setting.

## Supporting Information

S1 Checklist CONSORT Checklist. (DOC)

S1 Data Core.database.proces. (SAV)

S1 Protocol Trial study protocol. (PDF)

S1 Table Additional improvement actions, performed by the MFS and CFS departments. (DOC)
