# Supplementary material for: A Cluster-Randomized Trial of Two Strategies to Improve Antibiotic Use for Patients with a Complicated Urinary Tract Infection
Source: PLoS One. 2015 Dec 4;10(12):e0142672. doi: 10.1371/journal.pone.0142672 (PMC4670093; doi:10.1371/journal.pone.0142672)
Supplement: S1 Protocol — (PDF) [file pone.0142672.s003.pdf]

DEFINITIEF

Algemene gegevens / General Information

Programma / Programme

DoelmatigheidsOnderzoek: deelprogramma Implementatie

Subsidieronde / Subsidy round

IMP - round 09 - subsidieaanvraag

Projecttitel / Project title

(Cost)-Effectiveness of two different strategies to improve the quality of antibiotic use in patients with urinary tract infections (UTIs) in the hosptial

Is dit project een vervolg op een eerder project gehonoreerd door ZonMw? / Is this project a continuation of a project that has been previously funded bij ZonMw?

Nee

Aanvrager / Applicant

Dr. S.E. Geerlings

Functie / Position: | *Opleiding / Education:*

Studierichting / Subject:

T: 020-5664380 | *F:* 020-6972286 | *E:* S.E.Geerlings@amc.uva.nl

Academisch Medisch Centrum

Interne Kliniek

Meibergdreef 9

1105 AZ AMSTERDAM ZUIDOOST

Nederland

Projectleden / Project members

Dr. S.E. Geerlings (Projectleider en penvoerder)

Functie / Position: internist-infectioloog | *Opleiding / Education:*

Studierichting / Subject:

T: 020-5664380 | *F:* 020-6972286 | *E:* s.e.geerlings@amc.nl

Academisch Medisch Centrum

Inwendige Geneeskunde

Infectieziekten, Tropische Geneeskunde & AIDS

Meibergdreef 9

1105 AZ AMSTERDAM ZUIDOOST

Nederland

Prof. dr. L.J. Gunning-Schepers (Bestuurlijk verantwoordelijke)

Functie / Position: Voorzitter Raad van Bestuur | *Opleiding / Education:*

Studierichting / Subject:

T: 020-5662109 | *F:* | *E:* l.j.gunning@amc.nl

Academisch Medisch Centrum

Stafbureau Raad van Bestuur

Postbus 22660

DEFINITIEF

1100 DD AMSTERDAM ZUIDOOST
Nederland

Dr. R.B. Geskus (Mede aanvrager)
Functie / Position: statisticus | *Opleiding / Education:*
Studierichting / Subject:
T: 020-5666880 | *F:* | *E:* r.b.geskus@amc.nl
Academisch Medisch Centrum
Klinische Epidemiologie en Biostatistiek

Postbus 22660
1100 DD AMSTERDAM ZUIDOOST
Nederland

Prof. dr. R.P. Grol (Promotor)
Functie / Position: senior onderzoeker Kwaliteit van Zorg | *Opleiding / Education:*
Studierichting / Subject:
T: 024-3613129 | *F:* | *E:*
Universitair Medisch Centrum St. Radboud
Kwaliteit van Zorg
114 KWAZO
Postbus 9101
6500 HB NIJMEGEN
Nederland

Dr. M.E.J.L. Hulscher (Mede projectleider)
Functie / Position: Senior onderzoeker Kwaliteit van Zorg | *Opleiding / Education:*
Studierichting / Subject:
T: 024-3613129 | *F:* | *E:* m.hulscher@kwazo.umcn.nl
Universitair Medisch Centrum St. Radboud
Kwaliteit van Zorg

Postbus 9101
6500 HB NIJMEGEN
Nederland

Dr. B.C. Opmeer (Mede aanvrager)
Functie / Position: epidemioloog | *Opleiding / Education:*
Studierichting / Subject:
T: 020-5667002 | *F:* | *E:* b.c.opmeer@amc.nl
Academisch Medisch Centrum
Klinische Epidemiologie en Biostatistiek

Postbus 22660
1100 DD AMSTERDAM ZUIDOOST

DEFINITIEF

Nederland

Dr. M Prins (Mede aanvrager)
Functie / Position: arts-epidemioloog | *Opleiding / Education:*
Studierichting / Subject:
T: 020-5664380 | *F:* | *E:*
Academisch Medisch Centrum
Inwendige Geneeskunde
Infectieziekten, Tropische Geneeskunde & AIDS
Meibergdreef 9
1105 AZ AMSTERDAM ZUIDOOST
Nederland

Dr. J.M. Prins (Projectleider)
Functie / Position: internist-infectioloog | *Opleiding / Education:*
Studierichting / Subject:
T: 020-5664380 | *F:* 020-6972286 | *E:* j.m.prins@amc.nl
Academisch Medisch Centrum
Inwendige Geneeskunde
Infectieziekten, Tropische Geneeskunde & AIDS
Meibergdreef 9
1105 AZ AMSTERDAM ZUIDOOST
Nederland

Dr. J. Schouten (Mede aanvrager)
Functie / Position: internist | *Opleiding / Education:*
Studierichting / Subject:
T: 024-3613129 | *F:* | *E:* j.schouten@ic.umcn.nl
Universitair Medisch Centrum St. Radboud
Kwaliteit van Zorg

Postbus 9101
6500 HB NIJMEGEN
Nederland

Dr. T.M. de Reijke (Mede aanvrager)
Functie / Position: uroloog | *Opleiding / Education:*
Studierichting / Subject:
T: 020-5665779 | *F:* | *E:* t.m.dereyke@amc.nl
Academisch Medisch Centrum
Urologie

Meibergdreef 9
1105 AZ AMSTERDAM ZUIDOOST

Nederland

Samenwerking / Collaboration

Is er bij dit project sprake van samenwerking tussen onderzoek en praktijk? / Is there any collaboration between research and practice within this project?

Ja

Projectgegevens / Project information

Datum indienen (via ProjectNet) / Date of application

11-02-2008 20:51

Aandachtsgebieden / Focus

Themes:

Voorwaarden voor implementatie; Implementatiestrategieën en -processen ; Kosten en besparingen van implementatie; Combinatie;

Themes HTA methodology:

Projecttype / Project type

Implementatieproject

Samenvatting / Summary

DOELSTELLING/VRAAGSTELLING: Het beoordelen van de kosten-effectiviteit van twee strategieën om het antibioticagebruik voor patiënten met een urineweginfectie te verbeteren.
STUDIE OPZET/STUDIE POPULATIE/INTERVENTIE: Een cluster-randomized controlled trial, met opeenvolgende patiënten van afdelingen interne geneeskunde en urologie in 18 deelnemende ziekenhuizen. Na een voormeting in 50 patiënten op elke afdeling worden de ziekenhuizen gerandomiseerd tussen twee implementatiestrategieën. In een nameting zal op deze afdelingen de kwaliteit van het antibioticagebruik opnieuw gemeten worden.
IMPLEMENTATIESTRATEGIEËN: Een op theorie gebaseerde effectieve, maar arbeidsintensieve ‘state-of-the-art’ strategie wordt vergeleken met een populaire ‘public reporting’ strategie (openbaar maken van informatie over de kwaliteit van antibioticazorg).
UITKOMSTMATEN: Kwaliteit van antibioticagebruik wordt gemeten met indicatoren ontwikkeld uit een nationale richtlijn over de behandeling van gecompliceerde UWIs.
SAMPLE SIZE /DATA ANALYSE: Om een verschil van 15% in indicator score tussen de twee strategieën aan te tonen, met voor- en nametingen, alpha=0.05, power=0.80 en icc=0,10, moet een totaal aantal van: 2 (strategieën) x 2 (voor/nameting) x 18 clusters met 50 patiënten/cluster= 3,400 patiënten met een UWI geïncludeerd worden. Data worden uit de medische patiëntendossiers gehaald. Multilevel regression analysis wordt uitgevoerd om de effecten van de strategieën te vergelijken.
ECONOMISCHE EVALUATIE: De kosten van de twee verschillende implementatiestrategieën worden geëvalueerd in relatie tot de verschillen in effectiviteit.
TIJDPAD: Maand 1-10: Inclusie & voormetingen.Maand 11-23: Kwalitatieve analyse & implementatie activiteiten. Maand 24-30: Nametingen en voorbereiden data-analyse. Maand 30-36: Analyse en rapportage.

Summary
OBJECTIVE/RESEARCH QUESTION: To assess the (cost)-effectiveness of two strategies to improve the quality of antibiotic use in patients with urinary tract infections (UTIs).
STUDY DESIGN/POPULATION/INTERVENTION: A cluster-randomized controlled trial, including consecutive patients from two departments (Internal Medicine and Urology) in 18 participating hospitals.

After a baseline measurement in 50 patients from each department, using clinically validated indicators, hospitals will be randomized between two implementation strategies. In a post-intervention measurement, quality of antibiotic use will be assessed again for these departments.
IMPLEMENTATION STRATEGIES: A theory-based and effective, but labour-intensive strategy (the ‘state-of-the-art’ strategy) is compared to a currently popular strategy of providing public comparative information on quality of antibiotic care (the ‘public reporting’ strategy).
OUTCOME MEASURES: Clinically validated indicators extracted from a recent national guideline for the treatment of complicated UTIs.
SAMPLE SIZE/DATA ANALYSIS: To demonstrate a difference of 15% in indicator adherence between the two strategies, with baseline-and postintervention measurements, alpha=0.05, two-sided testing, power=0.80 and inter correlation coefficient (icc)=0,10, we need a total number of 2 (strategies) x 2 (pre/post-measurements) x 18 clusters with 50 patients/cluster= 3,400 patients with a UTI. Data will be extracted from medical charts of patients. Multilevel regression analyses will be performed to compare the effectiveness of both strategies.
ECONOMIC EVALUATION: The costs of the two different implementation strategies will be evaluated in relation to differences in effectiveness between the two strategies in improvement of quality indicators.
TIME SCHEDULE: Month 1-10: Patient inclusion and baseline measurements. Month 11-23: Qualitative analysis and implementation activities. Month 24-30: Post-intervention measurements and preparing data-analysis. Month 31-36: Data analysis and reporting.

Trefwoorden / Keywords

urinary tract infections, antibiotic use, implementation strategies, guidelines

Inhoud / Content

Probleemstelling / Problem definition

HEALTHCARE PROBLEM AND IMPLEMENTATION PROBLEM The use of antibiotics has led to a lower mortality and morbidity due to infectious diseases. However, resistance to antimicrobial drugs has increased since the first years of their clinical use. For example, in the treatment of patients with a urinary tract infection (UTI), also in the Netherlands increasing resistance rates of Escherichia coli to trimethoprim have been reported [1]. The total consumption of antibiotics is the main driving force [2]. Recently, it has been demonstrated that reducing antibiotic dispensing for UTIs at general-practice level is associated with reduced local antibiotic resistance rates of coliform isolates from urine samples [3]. Unnecessary use of antimicrobial agents may also lead to unnecessary high treatment costs. On the other hand, unjustified therapy with narrow-spectrum agents, not effectively treating the causative pathogen, can be equally detrimental [4]. So, optimal antibiotic use is considered relevant because of its impact on three key issues: clinical outcome, bacterial resistance and costs.To guarantee appropriate antibiotic use, treatment guidelines are developed. Adherence to guidelines improves clinical outcome [5,6]. Many (inter)national guidelines are now available for the antimicrobial treatment of patients with an infection. In the Netherlands, the Dutch Working Party on Antibiotic Policy (SWAB) formulates evidence-based guidelines for the antimicrobial treatment of the most relevant infections, including UTI. However, publication of a guideline does not guarantee its application. In a recent study in the USA it has been shown that the adherence to the Infectious Diseases Society of America (IDSA) guideline for the treatment of UTI was very poor [7]. Also in the Netherlands physicians can improve their practice in antibiotic prescription, referral and follow-up for UTIs [8,9]. For example, in a previous study on the quality of UTI care in four Dutch hospitals, we demonstrated a poor adherence to the SWAB guideline and a large room for improvement [9]. Therefore, implementation strategies are urgently needed to improve adherence to the guidelines.

The proposed study aims to get insight into the most cost-effective strategy to implement the Dutch SWAB guideline on UTI. In addition, the understanding of whether and why an implementation strategy is successful is still limited, since strategies are invariably found to be effective in some settings, but not in others [10]. With this study we will ascertain which elements of the strategy were particularly associated with successful implementation.

PATIENTS TARGETED IN THIS PROPOSAL Patients with complicated UTIs, treated at Urology- or Internal Medicine in- and outpatient departments. Together with pneumonia, (complicated) UTIs are in the hospital the most prevalent infectious diseases [11].

USUAL CARE Patients with a complicated UTI are mainly treated by Urologists or Internal Medicine specialists, working in the in- or outpatient clinic of the hospital.

COST-EFFECTIVENESS OF THE INTERVENTION/INNOVATION Inappropriate use of antibiotics leads to unnecessary recurrent infections, complications, associated health care utilisation and associated costs. Overall, improved antibiotic prescribing resulted in a reduction of both hospital acquired infections, costs and development of antimicrobial resistance [12, 13] (See Plan van Aanpak, ECONOMIC EVALUATION).

MOTIVATION FOR IMPLEMENTATION STRATEGIES In a previous ZonMw study (2300.0024) a multifaceted strategy to improve antibiotic use in hospitalised patients with lower respiratory tract infections (LRTI) was developed and tested for its effectiveness and feasibility [14] (see Plan van Aanpak STATE-OF-THE-ART STRATEGY). In the proposed study, this strategy will be fine-tuned and used to improve antibiotic use in patients with complicated UTIs. This ‘state-of-the-art’ strategy will be compared to a currently popular strategy of ‘public reporting’, i.e. collecting and publicly reporting comparative information about the cost and quality of health care in order to provide transparency on performance [15-17] (See Plan van Aanpak PUBLIC REPORTING STRATEGY). The literature, however, describes considerable uncertainty about the relative merits and risks of this major health policy initiative and additional research is required to determine whether a different approach (e.g. the state of the art strategy) would stimulate more improvement and whether the benefits of these programs outweigh their costs [18-21].

SEX, AGE OR CULTURAL BACKGROUND Patient (e.g. age, sex, co-morbidity, ethnic background), physician (e.g. urologist or internal medicine specialist, age, sex) or department (number of beds, specialised or not) related characteristics [7] might lead to differences in the choice of antimicrobial agents. Therefore, these factors will be included in our analyses.

Relevantie / Relevance

CHOSEN FOCUS/ HEALTH CARE PROBLEM RESOLUTION
To improve appropriate use of antibiotics in UTI, implementation of the SWAB guideline is necessary [22]. In our previous study on the quality of UTI care in four Dutch hospitals [9], which we can consider as a pilot study for this project proposal, we systematically developed quality indicators extracted from the national evidence-based SWAB guideline. These were subsequently tested in four different Dutch hospitals (4 Internal Medicine and 3 Urology departments). The indicators showed poor adherence to the guideline and a large room for improvement. The proposed study evaluates the cost-effectiveness of two strategies to improve adherence to the UTI guideline.

INCIDENCE OF THE TARGETED POPULATION UTIs occur frequently in people of all age groups, with an overall self-reported annual incidence of 12.1% among women and 3% among men. Thirty percent of the health care-associated infections are UTIs and nearly all of them can be considered complicated UTIs [11].

VARIATION IN USUAL CARE We showed that the current adherence to the guideline – measured by the indicators that we systematically developed from the guideline- showed a large variation. For example, performance of the 4 hospitals on the indicators: “Change catheter within 24 hours after initiating antibiotic treatment” and “Perform a urine culture” varied between (lowest performing department and best performing department) 14% and 33% and between 60% and 90% respectively [9].

CONTRIBUTION TO IMPLEMENTATION KNOWLEDGE
This study will contribute to our knowledge and understanding of implementation processes on four areas:
1) The proposed study will show us the most (cost)effective strategy to improve UTI antibiotic use in hospitals: the theory based and effective strategy (the ‘state-of-the-art’ strategy) or the adapted version of the currently popular strategy of providing public comparative information (the ‘public reporting’ strategy). At this moment health insurance companies and other involved parties are very interested in the measurement of quality of care and HCWs are forced to improve their quality of care without knowing the most effective method to do so.
2) The proposed study will add to our knowledge regarding the effectiveness of ‘public reporting’. The literature provides inconclusive evidence for this popular strategy [18]. To improve their acceptance, in the proposed study, comparative information on our UTI indicators will be made ‘public’ to all participating HCWs. Their experiences with this adapted version of public reporting will be measured and used to further develop a reporting method that is widely accepted by HCWs.
3) With regard to the ‘state-of-the-art’ strategy, we will be able to test whether a strategy that was developed for optimal antibiotic use in LRTI can -after fine tuning!- also be effective for optimal antibiotic use in UTIs.
4) Finally, to improve our understanding of whether and why an implementation strategy is successful, with this study we will evaluate which elements of the strategy were particularly associated with successful implementation [23,24], since it has been demonstrated that interventions are invariably found to be effective in some settings, but not in others [10, 25-28].

SIMILAR PROJECTS UNDERWAY To the best of our knowledge no similar studies have been reported or are under way [29].

RECENT REPORTS ON THE SUBJECT It was already described in the year 2000 in one of the recommendations of the report “Antibiotic Resistance” of the Dutch Health Council (Raad voor Gezondheidsonderzoek) [www.rgo.nl/publicaties/] that strategies to improve antibiotic use, in order to decrease the resistance development are urgently needed. Recently, more specific for the subject of this proposal, a call was published for a national performance not to treat asymptomatic bacteriuria [30].

POTENTIAL EFFECTS ON HEALTHCARE Adherence to the guideline will reduce the variation in care between the different professionals and hospitals. The results of an analysis of 60 studies, in which persuasive and restrictive methods to reduce unnecessary antibiotic use were evaluated, showed that interventions can improve antibiotic prescribing, resulting in a reduction of hospital acquired infections, related resource utilization, and development of antimicrobial resistance [13].

POTENTIAL EFFECTS ON COSTS A reduction in inappropriate use of antibiotics leads to a reduction of health care utilisation associated costs (following recurrences and complications). From a more long-term, societal perspective, increasing resistance rates and development of new antimicrobial drugs are very expensive. The state-of-the-art intervention is more labour-intensive and therefore more expensive. The proposed study will indicate for each strategy whether and at what threshold the

cost-effectiveness of the intervention will outweigh the implementation costs.

Kennisoverdracht, implementatie, bestendiging / Knowledge transfer, implementation, consolidation

The members of the project group have different specialties and backgrounds. This will lead to a comprehensive and multidisciplinary approach in the analysis of the clinical problem from the start of the project. After completion of the study, we will approach representatives of de Vereniging voor Infectieziekten (VIZ), Gezondheidsraad en Raad voor Volksgezondheid en Zorg (CEG), Nederlandse Vereniging voor Urologie (NVU), Nederlandse Vereniging van Ziekenhuis Apothekers (NVZA), Nederlandsche Internisten Vereniging (NIV), and Nederlandse Vereniging voor Medische Microbiologie (NVMM). These persons will be informed of the study results. As the results will be published in the medical literature, the findings can be applied by infectious diseases specialists and other Internal Medicine specialists, quality of care specialists, urologists, and medical microbiologists. Furthermore, we intend to present our results on the yearly symposium of the SWAB or when possible on a Health Care Insurance meeting. Before publication, the results will also be presented at national and international scientific meetings. In addition, patients for whom the results of the study may have clinical consequences, for example spinal cord injury patients with indwelling urinary catheters, will be informed by their associations. The members of the project group were involved in the writing of the national guidelines for the treatment of UTIs. When the results of this project will show that revision of the guideline is desired to improve its utility, the SWAB is willing to consider this revision.
As part of this project the representatives of the relevant departments of all participating hospitals (also the clusters randomized to the “public reporting strategy”) will receive in a feedback meeting the results of the process evaluations of both implementation strategies and the post-intervention measurements.

Doelstelling / Objective

In the proposed project we will compare two different implementation strategies in a cluster-randomized intervention study, to find out which implementation strategy is the most (cost)effective to improve the quality of antibiotic use for UTIs in the hospital.

Research questions:
1. What is the most (cost) effective strategy, the ‘state-of-the-art strategy’ or the ‘public-reporting strategy’, to improve the quality of antibiotic use in patients with complicated urinary tract infections (UTIs) in the hospital?
2. Which elements of the strategies were particularly associated with successful implementation?

Plan van aanpak / Strategy

EXPERIENCE WITH INTERVENTION AND IMPLEMENTATION ACTIVITIES
Dr. S. Geerlings and dr. J. Prins have written the national evidence-based SWAB guideline on the treatment of complicated UTIs [20]. Dr. J. Prins is board member of the SWAB and together with J. Schouten member of the Working Group for the development of Quality Indicators of the Dutch Associations of Internal Medicine Specialists (NIV). Dr. S. Geerlings is an ESCMID (European Society of Clinical Microbiology and Infectious Diseases) representative for the development of the IDSA guideline on catheter-related UTIs. Dr. M. Hulscher, dr. J. Schouten, and Prof. Dr. R. Grol are staff members of the Centre for Quality of Care Research of the Radboud University Nijmegen Medical Centre, which is internationally recognised for its expertise on and experience with implementation strategies in health care settings. They earlier successfully implemented interventions for the antimicrobial treatment of lower respiratory tract infections (LRTIs) [14,31].All group members were involved in the development and subsequent validation of the quality indicators for complicated UTIs [9]. Dr. M. Prins and Dr. R. Geskus are part of a infectious diseases epidemiological group which has a strong record in planning and evaluating public health interventions in the field of infectious diseases.

FOCUS OF IMPLEMENTATION The study aims at implementing the SWAB guideline on the treatment

of complicated UTIs in the hospital.

RELEVANT PARTIES INVOLVED The project group of this study includes both experts on antibiotic use and on implementation. In addition, relevant national groups support the study: the Dutch Associations of Internal Medicine Specialists (NIV) and the SWAB support the study.
Furthermore, both the Academic Medical Center and the Radboud University Nijmegen Medical Centre are academic hospitals and have close collaborations with a large number of other hospitals in their environment, regarding the education of medical doctors and different medical specialists, but also in the setting of research and recruitment of study patients (see deelnemende centra.pdf).

LIMITATIONS AND POSSIBILITIES OF THE STATE-OF-THE-ART-STRATEGY
Literature suggests that the choice of strategies to improve performance should be linked to the results of a problem analysis, i.e. the factors that facilitate or impede appropriate use of antibiotics. In our previous study (ZonMw 2300 0024) we performed an extensive analysis of factors relevant to the appropriate use of antibiotics in lower respiratory tract infections (as measured with the quality indicators) [14]. We used 18 semi-structured interviews and two group interviews with care providers (residents, physicians, nurses, microbiologists and clinical pharmacists) in 3 Dutch medium sized hospitals to qualitatively study and understand barriers to appropriate antibiotic use. Five indicators were discussed: the prescription of empirical antibiotic therapy that adheres to the guidelines, timely administration of antibiotics, adjusting antibiotic dosage to accommodate decreased renal function, switching and streamlining therapy, and blood and sputum culturing. Per recommendation barriers were classified into categories using a conceptual framework [23-25, 32] describing influencing factors at all possible levels (guideline, doctor, patient, system). Each recommendation elicited a different pattern of barriers. Based on this assessment of barriers, “targeted” implementation activities were developed and combined into a multifaceted implementation strategy.
For example, regarding the indicator ‘start empirical therapy adherent to the guideline’, physicians said that they WORRIED about patient outcome when prescribing narrow-spectrum antibiotic therapy. They DISAGREED WITH CURRENT GUIDELINE recommendations due to a lack of evidence justifying the recommendations and a lack of confidence in the guideline developers. They also LACKED INSIGHT into their own performance. External barriers were mainly related to the social context in which professionals operate: “OUT OF COURTESY TO COLLEAGUES, NO CRITICISM of the chosen antibiotic regimen is made at end-of-shift meetings”.
As a second example, regarding the ‘timelines of antibiotic administration’, most interviewees mentioned external barriers related to ORGANIZATIONAL FACTORS (e.g., substantial delays in delivering laboratory results to the department, antibiotics not present on the ward, IV drip not started). However, barriers were also created by a lack of the physician’s KNOWLEDGE about the impact that timely antibiotic administration can have on patient outcome and a LACK OF AGREEMENT with the guideline. Several (conflicting!) guidelines existed on the different wards. In addition is was stated that “ward nurses prioritize nonmedical issues (such as diet and social setting) during intake, leaving prescribed medication, including IV antibiotics, to the last or POSTPONING ADMINISTRATION until regular medication rounds”.
To improve KNOWLEDGE AND ATTITUDES on antibiotic evidence and guidelines, a key lecture on antibiotics (addressing all indicators) was given by a respected opinion leader.
To improve INSIGHT into their own performance, this opinion leader also provided feedback on indicator performance at the hospital level. This feedback included benchmarks at the hospital level and presented key issues for improvement. In addition, at an individual level, a 1-hour feedback and tutorial session was organized, in which small groups of peers compared their personal performances with respect to guideline adherence and discussed differences.
To INTEGRATE CONFLICTING WARD PROTOCOLS, representatives of all the relevant clinical specialties (physicians and nurses from the different wards, microbiologists and clinical pharmacists)

were asked to participate in developing a local consensus guideline based on the available evidence, leading to a clear and unequivocal critical-care pathway. This consensus care pathway was distributed to all doctors as a laminated, pocket version.
Journal clubs were organized to discuss controversies in the literature and guidelines on the preferred antibiotic management and indication of antibiotic use.
The process of ADMINISTRATING ANTIBIOTICS was analyzed to adapt intake procedures at the wards and the availability of antibiotics.
The implementation strategy was based on the results of the problem analysis (see also ‘Context analysis’) and comprised both a fixed part and a flexible part. The following implementation activities were performed:
In the first, fixed phase of the implementation period, hospitals installed a local organizing committee (LOC). In each hospital, a clinical pharmacist, a medical microbiologist, a physician, a pulmonologist, and a quality improvement officer participated. Hospitals received a key lecture given by a respected opinion leader at a kick-off meeting. Feedback on indicator performance at the hospital level was presented and provided in writing to all doctors treating hospital LRTIs. Feedback reports included benchmarks at the hospital level (best practice) and presented key issues for improvement. Consensus “critical-care pathways” were distributed to all doctors as a laminated, pocket version.
In the flexible phase, implementation activities were adjusted to the needs and wishes of every single hospital. At LOC meetings, local hospital baseline study results were discussed. The indicators most in need of improvement were given priority in the implementation protocol. Small groups of peers compared and discussed their personal performances. These activities were performed in three modules: initiation of therapy, changing therapy, and diagnostic procedures. If applicable, local processes of care were analyzed and work processes redesigned (e.g. regarding ‘timelines of antibiotic administration’, the process of administrating antibiotics was analyzed and improved). An external quality facilitator initiated and coordinated these activities.

Since most of our UTI indicators are the same as in those in this earlier LRTI project, we will proceed with the knowledge gained from this study. To ensure that our ‘state-of-the-art’ strategy focuses on the right set of barriers, as described above, an additional barrier inventory will be performed among HCWs who treat patients with complicated UTIs, to fine tune the strategy to fit UTI. In depth, semi structured interviews will be held with HCWs from various professional backgrounds and hospital settings (purposive sampling). Interviewees are asked to present a clinical case, e.g. the most recent patient with a complicated UTI who has been admitted in the 4 weeks preceding the interview. If no such patient can be found, the interviewer presents a previously prepared “dummy” patient before the interview. All sessions will be audiotaped. New interviews take place until no new information is gleaned.
All audiotaped interviews will be analysed either by using computer assisted qualitative data analysis software (ATLAS) or they will by transcribed verbatim and two researchers will independently review the manuscripts and mark comments about barriers to adherence. Remarks will be classified into categories of potential barriers.

LIMITATIONS AND POSSIBILITIES OF THE PUBLIC REPORTING STRATEGY
Advocates for public reporting argue that it could help providers to improve their performance by enabling them to benchmark their performance against other providers. Transparent performance data could help to hold them accountable. Others have expressed concern that the reliability of public reporting systems may be compromised by institutional variability in the definitions used or in the methods and resources used. To prevent the latter, in our project we use trained persons to collect identical information from all departments participating in the study.
It is assumed that making performance information available is an important step in stimulating HCWs to understand and improve their care process. The literature suggests that physicians are sceptical about public data and that they consider it to be of minimal use [18-21]. To improve their acceptance, in the

proposed study, comparative information on our UTI indicators will be made ‘public’ to all participating HCWs. It is important to stress that the responsibility to improve performance will be for the HCWs in the hospitals; in this study arm the study team plays no active role in this respect. Their experiences with this adapted version of public reporting will be measured and used to further develop a reporting method that is widely accepted by HCWs.

CHOSEN IMPLEMENTATION STRATEGIES & RELATION TO CURRENT INSIGHTS
Two implementation strategies are used in this project:
STATE-OF-THE-ART STRATEGY
We will fine tune an implementation strategy that was previously developed in accordance with the latest insights in effective implementation [10,25-27]. This strategy was developed in a previous ZonMw study (2300.0024) and turned out to be an effective strategy to improve some important indicators of optimal antibiotic use in hospitalised patients with LRTIs [14].

PUBLIC REPORTING STRATEGY
This ‘state-of-the-art’ strategy will be compared to an adapted version of a currently popular strategy of ‘public reporting’.

DESIGN The Urology- and Internal medicine departments in 18 hospitals (university, teaching, and non-teaching) will be cluster-randomized between the two different implementation strategies: The ‘state-of-the-art’ strategy and the ‘public reporting’ strategy. A cluster is a department in a hospital.

COLLECTION OF DATA AND STUDY POPULATION
The patients will be included at the departments of Urology and Internal Medicine. One local urologist and one internal medicine specialist will be responsible for the coordination of the study in each hospital. All these specialists have agreed to participate (see deelnemende centra.pdf) and they will form the Study Group of this project. The identification of patients will be made upon the national diagnosis registration system. Data will be collected retrospectively. Both in- and outpatients older than 16 years are eligible for inclusion when they were diagnosed and treated for a complicated UTI [9,22] between March 2008 and October 2009 (treatment for UTI not longer than one year before measurements) for the pre-intervention measurements and between December 2009 and June 2011 (in case of the “state-of-the-art-strategy” depending on the moment of the implementation of this strategy) for the post-intervention measurements.
A patient with an complicated UTI will be defined as a patient with a UTI with one of the following characteristics: any functional or anatomical abnormality of the urinary tract, pregnancy, immunocompromising disease or medication, male sex, or a UTI with symptoms of tissue invasion or systemic infection (pyelonephritis, urosepsis, prostatitis).
Based on the sample size calculation (see below) we will consecutively score 50 patient cases in each department from a certain starting point (March 2009), for the baseline measurements (phase 1), and the same number of consecutive patients from another starting point (December 2010) for the post-intervention measurements (phase 3). For all patients, clinical and laboratory data will be retrospectively extracted from clinical and outpatient medical records, admission sheets, nursing records, and medication charts. Trained persons will collect information from all departments participating in the study in a uniform way. Since all these procedures take place retrospectively, with the objective to optimalize patient care, it is not necessary to get informed consent of the patient. Still, we will notify the Medical Ethical Committee of the coordinating center (AMC Amsterdam) of our planned study and all data will be collected and entered in the database anonymously.

OUTCOME MEASURES AND PROCESS INDICATORS: We will use the same valid set of quality indicators measuring quality of antibiotic treatment for complicated UTIs in the hospital as described

before [9], and will measure the baseline performance of the four indicators, which turned out to be feasible for the Urology and Internal Medicine departments, namely:
1) Perform urine culture; 2) Prescribe treatment adherent to SWAB guideline; 3) Tailor treatment according to culture results; 4) Switch to oral treatment when possible.
After the implementation activities all indicators will be measured again in all participating departments (post intervention measurements), in the same manner as described above (Collection of data and Study population).

PROCESS EVALUATION MEASURES
To determine which elements of the strategies were particularly associated with successful implementation, for both strategies a process evaluation will be performed. Process evaluation will illuminate the mechanisms and processes responsible for the result and their variation within the departments. The actual exposure of the participants (i.e. the professionals at the departments) to the implementation activities, together with their experience of these activities may have influenced the final result (success or failure). Process evaluation is an important tool aimed at meticulously describing the actual exposure to the implementation activities (did the professionals participate in all sessions, did they use all the facilities offered etc), and the experience of the people exposed. This information is not only crucial for understanding the success –or lack of success- of implementation strategies, but also for providing basic data for economic evaluation of the improvement activities (see below).
Participation in the implementation activities as described is monitored closely, by documenting participation regarding all activities: e.g. who participated in the educational meetings, who participated in the feedback meeting, what was the number and duration of the different meetings, who read his/her individual feedback report etc. Data will be collected using a combination of data-collection methods, including questionnaires, systematic registration of time, minutes of meetings, attendance lists etc. This information will be related to effectiveness, to ascertain which elements of the strategy were particularly associated with successful implementation.
At the end of the study period, the experiences of study participants with the implementation activities will be measured using interviews and/or questionnaires. This information will be used to, if necessary, adapt the strategies to make them more acceptable and effective.

SAMPLE SIZE CALCULATION (MOTIVATE ASSUMPTIONS) AND FEASIBILITY OF RECRUITMENT
An indicator with an invariable high performance score does not discriminate quality of care between and within hospitals. Indicators with a median performance score of >85% will be defined as having little interdepartmental and interhospital variation and therefore considered to have little room for improvement. We consider a median performance score of < 60% as having enough room for improvement and intend to introduce the intervention programme in the “state-of-the-art” study arm (See Chosen implementation activities/strategies) when the median measured indicator score is < 60%. We already found in a pilot phase of this project that of the 4 indicators, which we will use, a score < 60% was found in respectively 2 and 3 indicators scored at the Internal Medicine- and Urology department [9]. It has been shown for pneumonia that the clinical indicator “Guideline adherence for empirical antibiotic therapy” (also one of our indicators) can be increased from 50.3% to 64.3% after implementation of an intervention programme [14]. We will consider a median difference of >15 % in scores of all indicators between the two implementation strategies after the interventions statistically significant. We will use all indicators, because the intervention might effect more than one indicator, not only those who scored < 60% at the baseline measurement. Multilevel regression analyses will be performed to compare the effectiveness of both strategies. The design has three levels of clustering: hospitals, department within hospitals and repeated measurements within individuals (i.e multiple indicators for each patient). We assume a difference of 15% between the two study arms in indicator adherence after the intervention (55% versus 70%). We have calculated the inter-cluster correlation (icc) from our own data [9] for 3 of the indicators which we will use and found a mean icc of 0.10. Using

alpha=0.05, two-sided testing, power=0.80 and icc=0.10, we need 18 clusters of 250 individuals per intervention if only one indicator is measured per individual. However, since we measure more indicators per individual, and assuming a correlation of 0.5 between the indicator values from the same individual, the number of individuals per clusters can be reduced by a factor five. This latter result follows from a sample size calculation for repeated measurements for binary outcomes. Hence, we effectively need 18 clusters of 50 individuals per intervention. This number is reasonable to recruit, since we included for every department participating in our earlier study between 90-100 patients with a UTI per department during one year [9]. At this moment, 18 hospitals have agreed to participate in this project (see deelnemende centra.pdf).

DATA ANALYSIS
Effectiveness of the two implementation strategies will be evaluated using multilevel linear regression analysis, adjusting for the hierarchical structure (patients within departments and departments within hospitals) in the data. In addition, we will explore whether the effect differs across subgroups at the patient (e.g. co-morbidity in patients), physician (i.e. specialisation of the physician), and department level (e.g. number of beds), and whether strategies are more successful for some indicators than for others.

ECONOMIC EVALUATION
There is little evidence regarding the efficiency of different strategies to disseminate and implement guidelines concerning the cost-effectiveness [10]. In the economic evaluation of implementation strategies, measures of benefit could be operationalized by patient outcomes or by intermediate end-points (process measures) reflecting the success of the implementation. If the intervention to be implemented has been demonstrated to be efficient, assessment by intermediate endpoints could suffice. If economic benefits are to be expected from specific changes in the health care process, patients where this change is observed can be documented, and differential costs can be incorporated by estimating the costs differences associated with this change.

The state-of –the-art intervention is more labour-intensive and therefore more expensive. Costs associated with each strategy are mainly driven by time spent by professionals, as well as by the research staff (researcher and quality of care specialist) dedicated to carry out all implementation activities, including preparation of educational materials, organizing meetings and courses, data acquisition (interviews, medical charts), data analyses, developing tailored programmes, implementing programmes, reassessment of all indicators, reanalyses. Costs of these activities (time, materials) will be estimated for each department and tailored programme, and offset to the observed effect of the implementation strategies in terms of the improvement of the score of the quality indicators.

On the other hand, increasing effectiveness of improved antibiotic use may influence the antibiotic regimen and hence could reduce costs of the antimicrobials and the length of hospital stay: e.g. an early intravenous (iv)-to-oral switch has been shown to be both cost-effective and safe in a selected group of patients [33]. For example, it has been shown that the switch from iv to oral treatment in more than half of the patients with one of the four common infections was unjustified delayed (mean delay switch was 5.1 days) [12], resulting in additional pharmacy and supplementary hospitalization costs. Decreasing resistance rates is also crucial from a long-term, societal perspective, as the development of new antimicrobial drugs is very expensive. However, guideline adherence may also generate additional costs, e.g. systematically requesting diagnostic or laboratory assessments. Differential costs associated with some indicators will be estimated, and incorporated in the cost-effectiveness analyses of the implementation strategies.

We identify the following phases in the implementation process:

Phase I: improving antibiotic use by education and behavior change of current nursing and medical professionals (including those in the current residency programs) in participating centers; and phase II: using the most cost-effective implementation strategy to improve antibiotic use in the Netherlands by education and behavior change.

The proposed study will effectuate the first phase, as we aim (in case of the “state of the art strategy”) to achieve a behavior change in current professionals to improve antibiotic use by a range of educational activities: distribution of educational materials, educational meetings, educational outreach visits, visits to local opinion leaders, development and implementation of audit and feedback reports, and reminders. The second phase would be conditional on the demonstrated effectiveness of the first phase, and can make use of the materials and experiences developed during the first phase. In the public reporting strategy, only measurement of performance generates costs.

We will estimate implementation costs associated with the implementation activities in the first phase, by documenting resource utilization associated with these activities. Based on these data implementation costs can be estimated at the organizational level (per department) as well as on the individual level (per patient). Furthermore, future costs associated with implementation in other Urology and Internal medicine department in the Netherlands will be estimated by extrapolating these estimates, evaluating the sensitivity for pertinent assumptions.

The economic evaluation will include the following analyses:
• comparison of implementation costs generated by the state-of-the art intervention and the public reporting strategy;
• assessment of the cost-effectiveness of the state-of-the art intervention as compared to the public reporting strategy by relating implementation costs to improvements in quality indicators and associated differential costs.
• estimating the incremental cost-effectiveness ratio expressed as the extra costs generated by the state-of-the art intervention as compared to the public reporting strategy, divided by the increase in the average performance of quality indicators.
• these economic analyses will be carried out at the overall level, as well as per department to allow for local differences in absolute and relative costs and effects of the two implementation strategies.
• estimating the total costs and clinical benefits associated with a nation-wide implementation using the best performing strategy in all remaining urology and internal medicine departments in the Netherlands;
• additional analyses will be carried out to evaluate the sensitivity of the results for assumptions or potential study-specific (location, motivation, etc) biases in the estimates.

SYTEMATIC REVIEW
The first step was to search whether comparable studies were done. We used different combinations of key words and searched in two databases (Pubmed and Cochrane Library) with different limit combinations (all search strategies see below) and scanned all titles and relevant abstracts. In conclusion, no studies were found in which implementation strategies for the improvement of the quality of care in the antimicrobial treatment of patients with a complicated UTI were investigated (See Table sytematic review for differences with the most comparable studies). The second and third steps were to discuss respectively the systematic reviews of Grimshaw et al. about guideline implementation and of Fung et al. about public reporting (see below).

1. For Tables see bijlage: Tables systematic review
In summary: no references were found, describing a study similar as in the current project proposal.

Keywords: urinary tract infections=#1 and guidelines = #2
Limits humans, English, Core Clinical Journals and Nursing Journals
Database Pubmed 136 ref, Cochrane Library 3 ref
Of 136 references, most 3 relevant references discussed in Table 1

Keywords: #2 and adherence
No limits
Database Pubmed 52 references, Cochrane library (0 ref), most 2 relevant references discussed in Table 2

Keywords: #2 and implementation
No limits
Database Pubmed 21 references, Cochrane library (0 ref), most 4 relevant references discussed in Table 3

Keywords: #1 and quality
Limits: humans, English, Core Clinical Journals and Nursing Journals, adults
Database Pubmed 60 references, Cochrane library (0 ref), no additional relevant references

Keywords: #2 and compliance
No limits
Database Pubmed 21 references, Cochrane library (0 ref), no additional relevant references

Keywords: #1 and quality indicators
No limits
Database Pubmed 22 references, Cochrane library (0 ref), no additional relevant references

Keywords: #1 and quality measurement
No limits
Database Pubmed 29 references, Cochrane library (0 ref), no additional relevant references

Keywords: antibiotic use and interventions
Limits: humans, English, Core Clinical Journals and Nursing Journals, adults, last 3 years because of
Database Pubmed 108 references, Cochrane review Davey P 2005 (see Table 4), 2 most relevant references mentioned in Table 4

2. Grimshaw, JM, Thomas RE, MacLennan G, Fraser C, Ramsay CR, Vale L, Whitty P, Eccles MP, Matowe L, Shirran L, Wensing M, Dijkstra R, Donaldson C. Effectiveness and efficiency of guideline dissemination and implementation strategies. Health Technol Ass 2004 Feb;8(6):iii-iv, 1-72.

Grimshaw et al. 2004 undertook a systematic review of the effectiveness (see below) and costs of different guideline development, dissemination and implementation strategies. The resource implications of these strategies were estimated, and a framework for deciding when it is efficient to develop and introduce clinical guidelines was developed.
SEARCH: STRATEGY, COMPARISON, OUTCOME
Single estimates of dichotomous process variables were derived for each study comparison based upon the primary end point or the median measure across several reported end points. Separate analyses were undertaken for comparisons of different types of intervention. The study also explored whether the effects of multifaceted interventions increased with the number of intervention components. Studies reporting economic data were also critically appraised. A survey to estimate the feasibility and likely

resource requirements of guideline dissemination and implementation strategies in United Kingdom settings was carried out with key informants from primary and secondary care.
Databases, selection procedure, methodological filters:
The authors searched MEDLINE, Healthstar, Cochrane Controlled Trial Register, EMBASE, SIGLE, and the specialized register of the Cochrane Effective Practice and Organization of Care (EPOC) group using the gold standard search strategy developed from handsearches of key journals (the EPOC strategy). In addition, the reviewers checked the reference lists of 51 systematic reviews of professional behaviour change strategies identified in the Effective Health Care bulletin on ‘Getting evidence into practice’ (1999; 5: 1-16)
Randomised controlled trials (RCT’s), controlled clinical trials (CCT’s), controlled before and after studies (CBA’s) and interrupted time series (ITS’s) were included, focusing on medically qualified healthcare professionals. Studies tested guideline dissemination and implementation strategies on objective measures of provider behaviour and/or patient outcomes. Methodological quality of the studies was measured, using the Cochrane EPOC group’s methodological quality criteria.
Results, manuscripts retrieved:
In total, 285 reports of 235 studies yielding 309 separate comparisons met the inclusion criteria; of these studies, 73 percent of comparisons evaluated multifaceted interventions, although the maximum number of replications of a specific multifaceted intervention was eleven comparisons. Reported details of the study interventions and contextual factors were poor and it was often difficult to assess the rationale for the choice of the intervention. There was little description of the potential barriers and facilitators to practice.
Overall, the majority of comparisons reporting dichotomous process data observed improvements in care; however, there was considerable variation in the observed effects both within and across interventions. Commonly evaluated single interventions were reminders, dissemination of educational materials, and audit and feedback. There were twenty-three comparisons of multifaceted interventions involving educational outreach. The majority of interventions observed modest (>5%, <= 10%) to moderate improvements (>10%, <= 20%) in care. No relationship was found between the number of component interventions and the effects of multifaceted interventions. Only 29.4 percent of comparisons reported any economic data. The majority of studies only reported costs of treatment; only twenty-five studies reported data on the costs of guideline development or guideline dissemination and implementation. The majority of studies used process measures for their primary end point, despite that only three guidelines were explicitly evidence-based (and may not have been efficient). Respondents to the key informant survey rarely identified existing budgets to support guideline dissemination and implementation strategies. In general, the respondents thought that only dissemination of educational materials and short (lunchtime) educational meetings were generally feasible within current resources.
Summary: There is an imperfect evidence base to support decisions about which guideline dissemination and implementation strategies are likely to be efficient under different circumstances. Decision-makers need to use considerable judgment about how best to use the limited resources they have for clinical governance and related activities to maximize population benefits. They need to consider the potential clinical areas for clinical effectiveness activities, the likely benefits and costs required to introduce guidelines and the likely benefits and costs as a result of any changes in provider behavior. Further research is required to develop and validate a coherent theoretical framework of health professional and organizational behavior and behavior change to inform better the choice of interventions in research and service settings and to estimate the efficiency of dissemination and implementation strategies in the presence of different barriers and effect modifiers.

3. Fung CH, Lim YW, Mattke S, Damberg C, Shekelle PG. Systematic review: the evidence that publishing patient care performance data improves quality of care. Ann Intern Med. 2008 Jan 15;148(2):111-23.

Fung et al. 2008 undertook a systematic review to synthesize the evidence for using publicly reported performance data to improve quality. Previous reviews have shown inconsistent effects of publicly reported performance data on quality of care, but many new studies have become available in the 7 years since the last systematic review.
Search & Data Sources: The authors performed an electronic search of Web of Science, MEDLINE, EconLit, and Wilson Business Periodicals (1999–2006) and an independent review of articles (1986–1999) identified in a previous systematic review. Only sources published in English were included.
Peer-reviewed articles assessing the effects of public release of performance data on selection of providers, quality improvement activity, clinical outcomes (effectiveness, patient safety, and patient-centeredness), and unintended consequences were selected.
The authors excluded opinion and theory articles, review articles, non–English-language articles, historical descriptions, and articles on awareness or comprehension of publicly reported performance data that did not also measure a change in the selection of providers, quality improvement activity, clinical outcomes, or unintended consequences.
Heterogeneity made comparisons across studies challenging. Only peer-reviewed, English-language articles were included.
Manuscripts retrieved & data extraction: The literature searches identified 2543 titles, from which they selected 143 articles for more detailed review. From these, 14 articles met the inclusion criteria. For articles published before 1999, they selected 18 of the 31 articles retrieved from Marshall and coauthors' review. They identified an additional 13 articles through review of reference lists or contacting experts. Thus, they identified 45 pertinent articles (of which 27 were not included in Marshall and coauthors' review) that evaluated the impact of public reporting on quality.
Data on study participants, reporting system or level, study design, selection of providers, quality improvement activity, outcomes, and unintended consequences were extracted from the studies selected.
Results: Many articles focus on a select few reporting systems. Synthesis of data from 8 health plan–level studies suggests modest association between public reporting and plan selection. Synthesis of 11 studies, all hospital-level, suggests stimulation of quality improvement activity. Review of 9 hospital-level and 7 individual provider–level studies shows inconsistent association between public reporting and selection of hospitals and individual providers. Synthesis of 11 studies, primarily hospital-level, indicates inconsistent association between public reporting and improved effectiveness. Evidence on the impact of public reporting on patient safety and patient-centeredness is scant.
Summary: Evidence is scant, particularly about individual providers and practices. Rigorous evaluation of many major public reporting systems is lacking. Evidence suggests that publicly releasing performance data stimulates quality improvement activity at the hospital level. The effect of public reporting on effectiveness, safety, and patient-centeredness remains uncertain.

TIME SCHEDULE (January 2009-January 2012)
Month 1-2: January -February 2009 Start of study, writing protocol, visiting all participating hospitals, planning data collection.
Month 3-10: March-October 2009: Phase 1: Baseline performance scoring of quality indicators & planning of feedback. Qualitative analysis to fine tune the state-of-the-art intervention.
Month 11-23: November 2009-November 2010: Phase 2: Qualitative analysis, and planning and implementation of different intervention strategies on all departments randomized to the ‘state-of-the art’ study arm, respectively reporting of the baseline results to the departments randomized to the ‘public reporting study arm’.
Month 24-30: December 2010-June 2011: Phase 3: Post-intervention measurements and preparing of data-analysis.
Month 31-36: July 2011-January 2012: Analysis and presenting of the data (see implementation), writing

the reports and implementation of the study results.

Expertise, voorgaande activiteiten en producten / Expertise, prior activities and products

Suzanne.E. Geerlings is an ID specialist and staff member at the Department of Infectious Diseases, Tropical Medicine and AIDS at the Academic Medical Center (AMC) in Amsterdam. The subject of her thesis was “Asymptomatic bacteriuria in women with diabetes mellitus: pathogenesis, risk factors and consequences.” She continued this line of research as a co-promotor and received two awards for it. At the moment she is the project leader of the project Non-antibiotic versus antbiotic prophylaxis for recurrent urinary tract infections (NAPRUTI), granted by ZonMw (number: 62000017/SGI12008) and is participating in other research projects mainly concerning UTIs. Furthermore, she is/was member of several working groups for the development of the revised and new guidelines for the treatment of urinary tract infections, those of the Dutch College of General Practioners (NHG); Dutch Working Party on Antibiotic Policy (SWAB) (main author), and Dutch Association of Urologists (NVU), and she is an ESCMID representative for the development of the IDSA guideline on catheter-related UTIs.

Jan M. Prins is an ID specialist and staff member at the Department of Infectious Diseases, Tropical Medicine and AIDS at the Academic Medical Center (AMC) in Amsterdam. He is in charge of the HIV outpatient clinic of the AMC, Amsterdam (1800 patients). His research lines Optimization of Antimicrobial Therapy, and Antiretroviral Therapy have resulted in many publications, including several RCTs, and he acted as 'copromotor' for several PhD theses in this field. He is head of the Infectious Diseases Fellowship Training Program at the AMC in Amsterdam. He is a board member of the Dutch Working Party on Antibiotic Policy (SWAB), chairs the SWAB guideline development committee, and chaired the working group for the development of the SWAB guideline for the treatment of complicated urinary tract infections. He is editor-in-chief of the Dutch national electronic antibiotic guide 'SWAB-ID' for use in hospitals.

Marlies E.J.L. Hulscher is a senior researcher at the Centre for Quality of Care Research (WOK). She has performed scientific research on determinants of and methods for the improvement of quality of care. Since 18 years she is involved in projects on quality of preventive care, infections and patient safety. Most studies include an analysis of barriers and facilitators, an effect evaluation (including the development of quality indicators) and a process evaluation to better understand the success or failure of the implementation strategy chosen. Examples of recent studies include ‘Prevention of antimicrobial resistance in hospitals: promoting appropriate use of antibiotics in hospital departments of internal and pulmonary medicine’ (ZonMw 2300.0024), ‘A model to improve the implementation of scientific advice on outbreak control measures (ZonMw 63000002) and Helping hands: comparing short-term and sustained effects of strategies to improve nurses’ adherence with hand hygiene prescriptions (ZonMw 80-007028-98-07101).

Prof.dr. Richard P.T.M. Grol is director of the Centre for Quality of Care Research (WOK). He has written over 300 peer reviewed papers and more than 20 books, mostly on issues related to quality in health care and the implementation of change. The Centre is internationally recognised for its expertise on and experience with implementation strategies in health care settings.

Brent Opmeer is a staff member of the Department of Clinical Epidemiology and Biostatistics in the Academic Medical Center in Amsterdam. He participated in the coordination of European collaborative studies (European Communities 4th Medical and Health Research Program COMAC; BIOMED1) concerning health services research and is since 1999 working in the department of Clinical Epidemiology and Biostatistics in the Academic Medical Centre. He has been involved in several evaluation studies (RCTs) in the fields of infectious diseases, surgery, and dermatology, especially focusing on the economic and methodological perspective. He has conducted a study on methods for determining patients' treatment preferences and trade-offs in health care technology assessment

research (ZONMW 2001-2003). He is currently involved in several studies in the field of obstetrics, gynaecology and reproductive medicine.

Theo de Reijke works as a urologist in the Academic Medical Centre. He graduated with a thesis on the role of immunotherapy in superficial bladder cancer. In 2006 he became the chairman of the EORTC-GU Group. Besides uro-oncology, one of Dr. De Reijke's main tasks is education and the development of CME programmes. He is head of the training programme for the residents at the AMC, coordinator of the oncology programme for the students at the AMC, chairman of the Dutch training programme for residents in urology, member of the examination committee of the European Board of Urology and member of the European Urology – Accredited Continuing Medical Education (EU-ACME) committee. At the Department of Urology he is responsible for patients presenting with UTIs and he is part of the project group investigating the role of UTI prevention using cranberries or lactobacillus compared to maintenance antiobiotics (NAPRUTI study).

Ronald B Geskus and Maria Prins have expertise in respectively the statistics and epidemiology of infections diseases and belong to a strong and multidisciplinary research group at the Amsterdam public health service, of whom M. Prins is the head.

For key references of all projectgroup members see Publications.

Publicaties / Publications

See References: 9, 14, 22, 24, 25, 26, 27, 31

Geerlings SE. Urinary tract infections in patients with diabetes mellitus: epidemiology, pathogenesis and treatment. Int J Antimicrob Agents. 2007 Dec 1

Meiland R, Stolk RP, Geerlings SE, ..., Hoepelman AI. Association between Escherichia coli bacteriuria and renal function in women: long-term follow-up.Arch Intern Med. 2007 Feb 12;167(3):253-7.

Meiland R, Geerlings SE, Stolk RP, Netten PM, Schneeberger PM, Hoepelman AIM. Asymptomatic bacteriuria in women with diabetes mellitus. Arch Intern Med 2006;166:2222-7.

Haaren van K, Visser HS, Vliet van S, Timmermans AE, Yadava R, Geerlings SE, Riet ter G, Pinxteren van B. NHG-Standaard Urineweginfecties (tweede herziening).Huisarts & Wetenschap. 2005;48(7):341-52.

SE Geerlings, R Meiland, IM Hoepelman. Antimicrobial treatment in diabetic women with asymptomatic bacteriuria. Letter. New Engl J Med 2003;348(10):957-58.

R Meiland, SE Geerlings, IM Hoepelman. Management of urinary tract infections in diabetes. Drugs. 2002;26(13):1859-68. Care 2002 Aug;25(8):1405-9.

Schouten JA, Hulscher MEJL, Wollersheim H, Braspenning J, Kullberg BJ, van der Meer JWM, Grol RPTM. Quality of antibiotic use for lower respiratory tract infections at hospitals: (how) can we measure it? Clinical Infectious Diseases 2005; 41: 450-460.

Schouten JA, Hulscher ME, Kullberg BJ, Cox A, Gyssens IC, van der Meer JW, Grol RP. Understanding variation in quality of antibiotic use for community-acquired pneumonia: effect of patient, professional and hospital factors. Journal of Antimicrobial Chemotherapy 2005; 56: 575-582.

Grol RPTM, Bosch MC, Hulscher MEJL, Eccles MP, Wensing M. Planning and studying improvement in

patient care. The use of theoretical perspectives. The Milbank Quarterly 2007; 85: 93-138.

Stone EG, Morton SC, Hulscher ME, Maglione MA, Roth EA, Grimshaw JM, Mittman BS, Rubenstein LV, Rubenstein LZ, Shekelle PG. Interventions that increase use of adult immunization and cancer screening services: a meta-analysis. Annals of Internal Medicine 2002; 136: 641-651.

Hulscher M, Wensing M, Grol R. Effectieve implementatie: theorieën en strategieën. ZON rapport. Den Haag, 2000.

Opmeer BC, El Moussaoui R, Bossuyt PM, Speelman P, Prins JM, de Borgie CA. Costs associated with shorter duration of antibiotic therapy in hospitalized patients with mild-to-moderate severe community-acquired pneumonia. J Antimicrob Chemother. 2007;60:1131-6.

Lindeboom JA, Kuijper EJ, Bruijnesteijn van Coppenraet ES, Lindeboom R, Prins JM. Surgical excision versus antibiotic treatment for nontuberculous mycobacterial cervicofacial lymphadenitis in children: a multicenter, randomized, controlled trial. Clin Infect Dis. 2007;44:1057-64.

van Vonderen MG, Gyssens IC, Hartwig NG, Kullberg BJ, Leverstein-van Hall MA, Natsch S, Prins JM. Optimalisation of the antibiotic policy in The Netherlands. XI. The national electronic antibiotic guide'SWAB-ID' for use in hospitals. Ned Tijdschr Geneeskd. 2006;150:2560-4. Dutch.

El Moussaoui R, Opmeer BC, de Borgie CA, Nieuwkerk P, Bossuyt PM, Speelman P, Prins JM. Long-term symptom recovery and health-related quality of life in patients with mild-to-moderate-severe community-acquired pneumonia. Chest. 2006;130:1165-72.

van Ruler O, Mahler CW, Boer KR, Reuland EA, Gooszen HG, Opmeer BC, de Graaf PW, Lamme B, Gerhards MF, Steller EP, van Till JW, de Borgie CJ, Gouma DJ, Reitsma JB, Boermeester MA; Dutch Peritonitis Study Group. Comparison of on-demand vs planned relaparotomy strategy in patients with severe peritonitis: a randomized trial.
JAMA. 2007;298(8):865-72.

Opmeer BC, Heydendael VM, De Borgie CA, Spuls PI, Bossuyt PM, Bos JD, De Rie MA.Costs of treatment in patients with moderate to severe plaque psoriasis:
economic analysis in a randomized controlled comparison of methotrexate and cyclosporine. Arch Dermatol. 2004;140(6):685-90.

Heydendael VM, Spuls PI, Opmeer BC, de Borgie CA, Reitsma JB, Goldschmidt WF, Bossuyt PM, Bos JD, de Rie MA. Methotrexate versus cyclosporine in moderate-to-severe chronic plaque psoriasis. N Engl J Med. 2003;349(7):658-65.

El Moussaoui R, de Borgie CA, van den Broek P, Hustinx WN, Bresser P, van den Berk GE, Poley JW, van den Berg B, Krouwels FH, Bonten MJ, Weenink C, Bossuyt PM,Speelman P, Opmeer BC, Prins JM. Effectiveness of discontinuing antibiotic treatment after three days versus eight days in mild to moderate-severe community acquired pneumonia: randomised, double blind study. BMJ. 2006;332(7554):1355.

H. Putter, M. Fiocco and R.B. Geskus. Tutorial in Biostatistics: Competing risks and multistate models. Statistics in Medicine 2007;26: 2389-2430.

Studer UE, Collette L, Whelan P, Albrecht W, Casselman J, de Reijke T, Knönagel H, Loidl W, Isorna S,

Sundaram SK, Debois M; the EORTC Genitourinary Group. Using PSA to Guide Timing of Androgen Deprivation in Patients with T0-4 N0-2 M0 Prostate Cancer not Suitable for Local Curative Treatment (EORTC 30891). Eur Urol. 2007 Dec 27; [Epub ahead of print]

Arentsen HC, de la Rosette JJ, de Reijke TM, Langbein S. Fluorescence in situ hybridization: a multitarget approach in diagnosis and management of urothelial cancer. Expert Rev Mol Diagn. 2007 7(1):11-9. Review.

Studer UE, Whelan P, Albrecht W, Casselman J, de Reijke T, Hauri D, Loidl W, Isorna S, Sundaram SK, Debois M, Collette L. Immediate or deferred androgen deprivation for patients with prostate cancer not suitable for local treatment with curative intent: European Organisation for Research and Treatment of Cancer (EORTC) Trial 30891. J Clin Oncol. 2006 20;24(12):1868-76.

Collette L, van Poppel H, Bolla M, van Cangh P, Vekemans K, Da Pozzo L, de Reijke TM, Verbaeys A, Bosset JF, Piérart M; European Organisation for Research and Treatment of Cancer (EORTC) Radiotherapy and Genito-urinary Groups. Patients at high risk of progression after radical prostatectomy: do they all benefit from immediate post-operative irradiation? (EORTC trial 22911). Eur J Cancer. 2005 41(17):2662-72. Epub 2005 Oct 11.

Ronald B Geskus, Maria Prins, Jean-Baptiste Hubert, Frank Miedema, Ben Berkhout, Christine Rouzioux, Jean-Francois Delfraissy, Laurence Meyer; The HIV RNA setpoint theory revisited, Retrovirology 2007; 4:65.

Ronald B. Geskus, Laurence Meyer, Jean-Baptiste Hubert, Hanneke Schuitemaker, Ben Berkhout, Christine Rouzioux, Ioannis D. Theodorou, Jean-Francois Delfraissy, Maria Prins and Roel A.Coutinho; Causal pathways of the effects of age and the CCR5-delta32, CCR2-64I and SDF-1 3'A alleles on AIDS development, JAIDS 2005; 39: 321-326.

Ronald B. Geskus, Frank A. Miedema, Jaap Goudsmit, Peter Reiss, Hanneke Schuitemaker and Roel A. Coutinho; Prediction of Residual Time to AIDS and Death Based on
Markers and Cofactors, JAIDS, 32: 514-521.

R.B. Geskus; Methods for estimating the AIDS incubation time distribution when date of seroconversion is censored, Statistics in Medicine 2001; 20(5): 795-812

van den Berg C, Smit C, van Brussel G, Coutinho R, Prins M. Full participation in harm reduction programs associated with a decreased risk for HIV and HCV: evidence from the Amsterdam Cohort Studies among drug users. Addiction 2007:102:1454-62

Smit C, Geskus R, Walker S, Sabin C, Coutinho R, Porter K, Prins M; for the CASCADE Collaboration. Effective therapy has altered the spectrum of cause-specific mortality following HIV seroconversion. AIDS. 2006;20:741-749.

Referenties / References

1. Neeling de AJ, Verbrugh HA.Consumption of antimicrobial agents and antimicrobial resistance among medically important bacteria in the Netherlands.Nethmap 2007. See: http://www.swab.nl
2. Goossens H, et al Outpatient antibiotic use in Europe and association with resistance: a cross-national database study.Lancet. 2005 Feb 12-18;365(9459):579-87.
3. Butler CC, et al. Containing antibiotic resistance: decreased antibiotic-resistant coliform urinary tract

infections with reduction in antibiotic prescribing by general practices. Br J Gen Pract 2007;57:785-92.
4. Kollef MH. Containing antibiotic resistance: decreased antibiotic-resistant coliform urinary tract infections with reduction in antibiotic prescribing by general practices.Clin Infect Dis 2000 September;31 Suppl 4:S131-8.
5. Menendez R et al. Guidelines for the treatment of community-acquired pneumonia: predictors of adherence and outcome.Am J Respir Crit Care Med. 2005 Sep 15;172(6):757-62. Epub 2005 Jun 3.
6. Frei CR et al. Impact of guideline-concordant empiric antibiotic therapy in community-acquired pneumonia. Am J Med. 2006 Oct;119(10):865-71.
7. Taur Y, Smith MA. Adherence to the Infectious Diseases Society of America guidelines in the treatment of uncomplicated urinary tract infection.Clin Infect Dis 2007;44:769-74.
8. Harmsen M et al. Management of children's urinary tract infections in Dutch family practice: a cohort study.BMC Fam Pract. 2007 Mar 13;8-9.
9. Hermanides HS, Hulscher MEJL, Schouten JA, Prins JM and Geerlings SE. Development of quality indicators for the antibiotic treatment of complicated urinary tract infections in the hospital. Clin Infect Dis March 1, 2008, In press.
10. Grimshaw JM et al. Effectiveness and efficiency of guideline dissemination and implementation strategies. Health Technol Ass 2004 Feb;8(6):iii-iv, 1-72.
11. Foxman B, Brown P. Epidemiology of urinary tract infections: transmission and risk factors, incidence, and costs.Infect Dis Clin N Am June 2003,17: 227-41.
12. Di Giammarino L et al. Evaluation of prescription practices of antibiotics in a medium-sized Swiss hospital.Swiss Med Wkly. 2005 Dec 10;135(47-48):710-4.
13. Davey P et al. Cochrane Database of Systematic Reviews 2005, Issue 4. Art. No.: CD003543. DOI: 10.1002/14651858.CD003543.pub2
14. Schouten JA et al.Tailored interventions to improve antibiotic use for lower respiratory tract infections in hospitals: a cluster-randomized, controlled trial. Clin Infect Dis. 2007 Apr 1;44(7):931-41.
15. Gezondheidsraad /Raad voor de Volksgezondheid & Zorg. Vertrouwen in verantwoorde zorg? Effecten van en morele vragen bij het gebruik van prestatie-indicatoren. Signalering ethiek en gezondheid 2006/1, Den Haag: Centrum voor ethiek en gezondheid, 2006.
16. Marshall MN et al. The public release of performance data: what do we expect to gain? A review of the evidence. JAMA. 2000 Apr 12;283(14):1866-74.
17. Wollersheim H, Faber M, Grol R. Does the public reporting of quality of care indicators improve the performance of health care providers and institutions? A systematic review of the evidence. Submitted for publication.
18. Lindenauer PK et al. Public reporting and pay for performance in hospital quality improvement. New Engl J Med 2007;356(5):486-96.
19. Colmers, JM. Public reporting and transparency. The Commonwealth Fund, January 2007, www.cmwf.org
20. McKibben L, Horan T, Tokars JI, Fowler G, Cardo DM, Pearson ML, Brennan PJ.Guidance on public reporting of Healthcare-associated infections: recommendations of the Healthcare Infection ControlPractices Advisory Committee Am J infect control 2005; 33:217-226.
21. Fung CH, Lim YW, Mattke S, Damberg C, Shekelle PG. Systematic review: the evidence that publishing patient care performance data improves quality of care. Ann Intern Med. 2008 Jan 15;148(2):111-23.
22. Geerlings SE, …, Prins JM. Optimisation of the antibiotic policy in the Netherlands. X. The SWAB guideline for antimicrobial treatment of complicated urinary tract infections. Ned Tijdschr Geneeskd. 2006 Oct 28;150(43):2370-6. http://www.swab.nl
23. Kanouse D et al. Dissemination of effectiveness and outcomes research.Health Policy 1995; 34: 167-92.
24. Hulscher MEJL, Laurant MGH, Grol RPTM. Process evaluation on quality improvement interventions.Qual Saf Health Care 2003; 12: 40-46.

25. Grol R. Personal paper. Beliefs and evidence in changing clinical practice.BMJ 1997; 315 (7105): 418-21.
26. Grol R, Grimshaw J. From best evidence to best practice: effective implementation of change in patients' care.The Lancet 2003; 362: 1225-30.
27. Grol R. Successes and failures in the implementation of evidence-based guidelines for clinical practice.Med Care. 2001 Aug;39(8 Suppl 2):II46-54.
28. Oxman AD. No magic bullets. London: North Thames Regional Health Authority, 1994.
29. http://www.controlled-trials.com/mrCT/
30. Gross PA, Patel B. Reducing antibiotic overuse: a call for a national performance measure for not treating asymptomatic bacteriuria. Clin Infect Dis 2007;45:1335-7.
31. Schouten JA, Hulscher ME, ……., Grol RP. Barriers to optimal antibiotic use for community-acquired pneumonia at hospitals: a qualitative study.Qual Saf Health Care. 2007 Apr;16(2):143-9.
32. Cabana MD et al. Why don't physicians follow clinical practice guidelines? A framework for improvement. JAMA 1999;282(15): 1458-65.
33. Rhew DC et al. Early switch and early discharge strategies in patients with community-acquired pneumonia: a meta-analysis. Arch Intern Med. 2001 Mar 12;161(5):722-7.

Financiële gegevens / Financial data

Geplande duur in maanden / Planned duration in months

36 maanden / months

ZonMw budget

	Jaar / Year								
Kostenpost / Cost item	*1*	*2*	*3*	*4*	*5*	*6*	*7*	*8*	*Totaal / Total*
Personeel	110.511	138.881	127.355	0	0	0	0	0	376.747
Materieel	750	750	750	0	0	0	0	0	2.250
Implementatie	0	0	3.000	0	0	0	0	0	3.000
Apparatuur	1.500	1.500	1.500	0	0	0	0	0	4.500
Overig	4.000	4.500	5.450	0	0	0	0	0	13.950
**Totaal / Total**	**116.761**	**145.631**	**138.055**	**0**	**0**	**0**	**0**	**0**	**400.447**

Co-financiering / Cofinancing

Naam co-financier / Name of cofinancier	Bedrag / Amount	Status

Bijzondere gegevens / Additional information

Vergunningen / Permits

	Vergunning nodig / Permit required?		Vergunning verkregen / Permit obtained?	
	Ja / Yes	Nee / No	Ja / Yes	Nee / No

DEFINITIEF

METC/DEC		X		X
WBO		X		X
Biohazards		X		X

Andere vergunningen / Other permits

Historie subsidieaanvraag / History grant application

Deze aanvraag is eerder ingediend bij het programma / This grant application has previously been submitted to the ZonMw programme:

Projectnummer / Project number:

Deze aanvraag is ook bij andere organisaties dan ZonMw ingediend / This grant application has also been submitted to other organizations than ZonMw:

Ondertekening / Signatures

Naam penvoerder-projectleider:<br><br>S.E. Geerlings	Naam bestuurlijk verantwoordelijke:<br><br>L.J. Gunning-Schepers
Plaats en datum:	Plaats en datum:
Handtekening:<br><br>--	Handtekening:<br><br>--

1.a PERSONEELSKOSTEN

jaar 1

nr.	Functie	Schaal	Maand salaris	Aantal maanden	Formatie	Bruto salaris	Raming CAO-wijz. 4%	Werkgevers-bijdragen 37%	Subtotaal	Opslag op personeels-kosten 16%	Totaal
1	Arts-onderzoeker	10,2	2.519	12	1,00	30.228	31.437	11.632	43.069	6.891	49.960
2	Senior Onderzoeker staf AN	UMS/0	6.667	12	0,10	8.000	8.320	3.079	11.399	1.824	13.223
3	Senior Onderzoeker	13,4	4.759	12	0,20	11.422	11.878	4.395	16.273	2.604	18.877
4	Trial Nurse	8,5	2.648	12	0,40	12.710	13.219	4.891	18.110	2.898	21.007
5	Datamanager	12,0	3.753	12	0,10	4.504	4.684	1.733	6.417	1.027	7.443
	Totaal				1,80	66.864	69.539	25.729	95.268	15.243	110.511

1.b PERSONEELSKOSTEN jaar 2

nr.	Functie	Schaal	Maand salaris	Aantal maanden	Formatie	Bruto salaris	Raming CAO-wijz. 6%	Werkgevers-bijdragen 37%	Subtotaal	Opslag op personeels-kosten 16%	Totaal
1	Arts-onderzoeker	10,3	2.648	12	1,00	31.776	33.683	12.463	46.145	7.383	53.528
2	Senior Onderzoeker staf AN	UMS/1	7.011	12	0,10	8.413	8.918	3.300	12.218	1.955	14.172
3	Senior Onderzoeker	13,5	4.895	12	0,20	11.748	12.453	4.608	17.060	2.730	19.790
4	Trial Nurse	8,6	2.704	12	0,80	25.958	27.516	10.181	37.697	6.031	43.728
5	Datamanager	12,1	3.863	12	0,10	4.636	4.821	1.784	6.605	1.057	7.662
	Totaal				2,20	82.531	87.390	32.334	119.725	19.156	138.881

1.c PERSONEELSKOSTEN jaar 3

nr.	Functie	Schaal	Maand salaris	Aantal maanden	Formatie	Bruto salaris	Raming CAO-wijz. 8%	Werkgevers-bijdragen 37%	Subtotaal	Opslag op personeels-kosten 16%	Totaal
1	Arts-onderzoeker	10,4	2.763	12	1,00	33.156	35.808	13.249	49.058	7.849	56.907
2	Senior Onderzoeker staf AN	UMS/2	7.355	12	0,10	8.826	9.532	3.527	13.059	2.089	15.148
3	Senior Onderzoeker	13,6	5.031	12	0,20	12.074	13.040	4.825	17.865	2.858	20.724
4	Trial Nurse	8,7	2.763	12	0,40	13.262	14.323	5.300	19.623	3.140	22.763
5	Datamanager	12,2	3.971	12	0,15	7.148	7.434	2.750	10.184	1.629	11.814
	Totaal				1,85	74.467	80.138	29.651	109.789	17.566	127.355

1	Arts-onderzoeker	1,00	160.395
2	Senior Onderzoeker staf AMC	0,10	42.544
3	Senior Onderzoeker	0,20	59.391
4	Trial Nurse	0,53	87.498
5	Datamanager	0,12	26.919
	Totale kosten	1,83	376.747

**Materiele kosten**	**aantal**	**prijs**	**kosten jaar 1**	**kosten jaar 2**	**kosten jaar 3**	**kosten jaar 4**	**kosten totaal**
1 Materiaal			750	750	750		2.250
2			0	0	0		0
3			0	0	0		0
subtotaal			750	750	750		2.250

**Communicatie en Implementatiekosten**	**kosten jaar 1**	**kosten jaar 2**	**kosten jaar 3**	**kosten jaar 4**	**kosten totaal**
1 Meeting deelnemende ZHen voor presentatie resultaten	0	0	3.000		3.000
2	0	0	0		0
3	0	0	0		0
subtotaal	0	0	3.000		3.000

**Apparatuurkosten**	**Aanschaf**	**kosten jaar 1**	**kosten jaar 2**	**kosten jaar 3**	**kosten jaar 4**	**kosten totaal**
1 laptop/printer	5.000	1.500	1.500	1.500		4.500
subtotaal		1.500	1.500	1.500		4.500

**Overige kosten**	**kosten jaar 1**	**kosten jaar 2**	**kosten jaar 3**	**kosten jaar 4**	**kosten totaal**
1 Congreskosten	2.000	2.000	2.000		6.000
2 Drukkosten proefschrift			1.200		1.200
3 Reiskosten research nurse	2.000	2.500	2.000		6.500
4 Accountantskosten			250		250
subtotaal	4.000	4.500	5.450		13.950

**Totaal niet-personele kosten**	**kosten jaar 1**	**kosten jaar 2**	**kosten jaar 3**	**kosten jaar 4**	**kosten totaal**
Materiele kosten	750	750	750		2.250
Communicatie en Implementatiekosten	0	0	3.000		3.000
Apparatuurkosten	1.500	1.500	1.500		4.500
Overige kosten	4.000	4.500	5.450		13.950
Totaal	6.250	6.750	10.700		23.700

ZonMw Doelmatigheid 2009

Begroting Research project AMC

Title : (Cost)- effectiveness of two different strategies to improve the quality of antibiotic use in patients with urinary tract infections (UTIs) in 17 Dutch hospitals

Projectleader: Suzanne Geerlings

Periode 1-1-2009 tm 1-1-2012

	Description	year 1	year 2	year 3	Total
1	Personnel	110.511	138.881	127.355	376.747
2	Material costs	750	750	750	2.250
3	Communication and Implementation	0	0	3.000	3.000
4	Hardware	1.500	1.500	1.500	4.500
5	Miscellaneous	4.000	4.500	5.450	13.950
	Total	116.761	145.631	138.055	**400.447**

Eigen bijdrage van het AMC 0,2 fte stafleden	€ 26.436	€ 28.285	€ 30.210	**€ 84.931**

Tables of Sytematic review behorende bij project: (Cost)-Effectiveness of two different strategies to improve the quality of antibiotic use in patients with urinary tract infections (UTIs) in the hosptial, projectnummer: 80-82315-98-09004

Table 1

**Reference**	**Summary**	**New in current project proposal**
Grover ML et al. Assessing adherence to evidence-based guidelines for the diagnosis and management of uncomplicated urinary tract infection. Mayo Clin Proc. 2007 Feb;82(2):181-5.	To assess adherence to evidence-based guidelines for the diagnosis and management of uncomplicated urinary tract infection (UTI) in a family medicine residency clinic setting.	Complicated UTI. Quality indicators. Comparing two implementation strategies.
Cohen AL et al. Compliance with guidelines for the medical care of first urinary tract infections in infants: a population-based study. Pediatrics. 2005 Jun;115(6):1474-8.	To describe the medical care of children in their first year of life after a first urinary tract infection	Adults. Comparing two implementation strategies.
Flottorp S et al. Cluster randomised controlled trial of tailored interventions to improve the management of urinary tract infections in women and sore throat. BMJ. 2002 Aug 17;325(7360):367.	To assess the effectiveness of tailored interventions to implement guidelines for urinary tract infections in women and sore throat.	In the hospital. Comparing two implementation strategies

Table 2

**Reference**	**Summary**	**New in current project proposal**
Harmsen M et al. Management of children's urinary tract infections in Dutch family practice: a cohort study. BMC Fam Pract. 2007 Mar 13;8:9.	To describe the clinical management of young children's UTIs in Dutch primary care and to compare this to the national guideline recommendations.	Adults. In the hospital. Comparing two implementation strategies.
Taur Y et al. Adherence to the Infectious Diseases Society of America guidelines in the treatment of uncomplicated urinary tract infection. Clin Infect Dis. 2007 Mar 15;44(6):769-74. Epub 2007 Feb 1.	1) examine the prescribing practices for the treatment of uncomplicated UTI and 2) determine whether these practices were influenced by the recommendation in the Infectious Diseases Society of America guidelines	Complicated UTI. Comparing two implementation strategies.

Table 3

**Reference**	**Summary**	**New in current project proposal**
Ali MH et al. Failure to implement hospital antimicrobial prescribing guidelines: a comparison of two UK academic centres. J Antimicrob Chemother. 2006 May;57(5):959-62. Epub 2006 Mar 10.	To investigate (i) which antimicrobial drugs were chosen by hospital doctors faced with two common infections [community-acquired pneumonia (CAP) and urinary tract infection (UTI)], (ii) whether these choices were compliant with local guidance and (iii) the factors that influenced antimicrobial choice.	Quality indicators. Comparing two implementation strategies.
Rautakorpi UM et al. The Antimicrobial Treatment Strategies (MIKSTRA) program: a 5-year follow-up of infection-specific antibiotic use in primary health care and the effect of implementation of treatment guidelines. Clin Infect Dis. 2006 May 1;42(9):1221-30. Epub 2006 Mar 30.	Moderate qualitative improvements in antibiotic use were observed after multifaceted intervention, but prescribing for unjustified indications did not decrease. Obtained infection-specific information on management of patients with infections in primary health care is an important basis for planning targeted interventions in the future.	In the hospital. Quality indicators. Comparing two implementation strategies.
Flottorp S et al. Process evaluation of a cluster randomized trial of tailored interventions to implement guidelines in primary care--why is it so hard to change practice? Fam Pract. 2003 Jun;20(3):333-9.	Evaluation how the interventions were received and to understand why practices did or did not change.	In the hospital. Comparing two implementation strategies
Stuart ME et al. Successful implementation of an evidence-based clinical practice guideline: acute dysuria/urgency in adult women. HMO Pract. 1997 Dec;11(4):150-7.	This paper describes the development and successful implementation of an evidence-based clinical practice guideline dealing with uncomplicated urinary tract infection in adult women (acute dysuria guideline) with a combination of implementation strategies.	Complicated urinary tract infections. Quality indicators. Comparing two implementation strategies.

Table 4

**Reference**	**Summary**	**New in current project proposal**
Metlay JP et al. Cluster-randomized trial to improve antibiotic use for adults with acute respiratory infections treated in emergency departments. Ann Emerg Med. 2007 Sep;50(3):221-30. Epub 2007 May 23.	To evaluate the effectiveness of an educational program in hospital emergency departments (EDs) targeting reduction in antibiotic overuse for acute respiratory tract infections.	Urinary tract infections. Quality indicators. Comparing two implementation strategies.
Loeb M et al. Effect of a multifaceted intervention on number of antimicrobial prescriptions for suspected urinary tract infections in residents of nursing homes: cluster randomised controlled trial. BMJ. 2005 Sep 24;331(7518):669. Epub 2005 Sep 8.	To assess whether a multifaceted intervention can reduce the number of prescriptions for antimicrobials for suspected urinary tract infections in residents of nursing homes.	In the hospital. Comparing two implementation strategies.
Davey P, Brown E, Fenelon L, Finch R, Gould I, Hartman G, Holmes A, Ramsay C, Taylor E, Wilcox M, Wiffen P. Interventions to improve antibiotic prescribing practices for hospital inpatients. *Cochrane Database of Systematic Reviews* 2005, Issue 4. Art. No.: CD003543. DOI: 10.1002/14651858.CD003543.pub2.	The primary aim is to identify interventions that alone, or in combination, are effective in improving antibiotic prescribing to hospital inpatients.	Comparing two implementation strategies. Only complicated UTIs.

onze lieve vrouwe gasthuis

oosterpark 9 / prinsengracht 769
postbus 95500, 1090 HM amsterdam
telefoon (020) 599 91 11
www.olvg.nl

Programmasecretaris ZonMw
Doelmatigheidsonderzoek, deelprogramma Implementatie
Geertje Appel
Postbus 93243
2509 AE Den Haag
doelmatigheidsonderzoek@zonmw.nl

olvg

22 januari 2008

Betreft: Participatie aan project (Cost)-Effectiveness of two different strategies to improve the quality of antibiotic use in patients with urinary tract infections (UTIs) in the hosptial, projectnummer: 80-82315-98-09004

Geachte mevrouw Appels,

Hierbij verklaren wij dat wij als deelnemend centrum willen participeren aan bovenstaande studie. De projectleiders (Dr. S.E. Geerlings of Dr. J.M. Prins) hebben ons uitgelegd wat deelname voor ons inhoudt.

Met vriendelijke groet,

Namens de afdeling Interne Geneeskunde
Prof. dr. K. Brinkman

Namens de afdeling Urologie:
Dr. G. van Andel

Ziekenhuis Amstelland

Programmasecretaris ZonMw
Doelmatigheidsonderzoek, deelprogramma Implementatie
Geertje Appel
Postbus 93243
2509 AE Den Haag
doelmatigheidsonderzoek@zonmw.nl

24 Januari 2008

Betreft: Participatie aan project (Cost)-Effectiveness of two different strategies to improve the quality of antibiotic use in patients with urinary tract infections (UTIs) in the hosptial,
projectnummer: 80-82315-98-09004

Geachte mevrouw Appels,

Hierbij verklaren wij dat wij als deelnemend centrum willen participeren aan bovenstaande studie. De projectleiders (Dr. S.E. Geerlings of Dr. J.M. Prins) hebben ons uitgelegd wat deelname voor ons inhoudt.

Met vriendelijke groet,

Naam ziekenhuis…Amstelland………………………….

Te…………………Amstelveen………………………

Namens de afdeling Interne geneeskunde:	Namens de afdeling urologie:
Naam: ………………………………………	Naam:……Dr. Y. Reisman……………………….
Handtekening: ………………………………	Handtekening:

HagaZiekenhuis

Programmasecretaris ZonMw
Doelmatigheidsonderzoek, deelprogramma Implementatie
Geertje Appel
Postbus 93243
2509 AE Den Haag
doelmatigheidsonderzoek@zonmw.nl

30 Januari 2008

Betreft: Participatie aan project (Cost)-Effectiveness of two different strategies to improve the quality of antibiotic use in patients with urinary tract infections (UTIs) in the hosptial,
projectnummer: 80-82315-98-09004

Geachte mevrouw Appels,

Hierbij verklaren wij dat wij als deelnemend centrum willen participeren aan bovenstaande studie. De projectleiders (Dr. S.E. Geerlings of Dr. J.M. Prins) hebben ons uitgelegd wat deelname voor ons inhoudt.

Met vriendelijke groet,

Naam ziekenhuis HagaZiekenhuis

Te Den Haag

Namens de afdeling Interne geneeskunde:

Naam: Dr R.M. Valentijn

Handtekening: ....

Namens de afdeling urologie:

Naam: F.M.J.A. Froeling

Handtekening: ....

Rode Kruis *ziekenhuis*

Programmasecretaris ZonMw
Doelmatigheidsonderzoek, deelprogramma Implementatie
Geertje Appel
Postbus 93243
2509 AE Den Haag
doelmatigheidsonderzoek@zonmw.nl

30.. Januari 2008

Betreft: Participatie aan project (Cost)-Effectiveness of two different strategies to improve the quality of antibiotic use in patients with urinary tract infections (UTIs) in the hosptial, projectnummer: 80-82315-98-09004

Geachte mevrouw Appels,

Hierbij verklaren wij dat wij als deelnemend centrum willen participeren aan bovenstaande studie. De projectleiders (Dr. S.E. Geerlings of Dr. J.M. Prins) hebben ons uitgelegd wat deelname voor ons inhoudt.

Met vriendelijke groet,

Naam ziekenhuis......Rode Kruis Ziekenhuis

Te..........Beverwijk..........................

Namens de afdeling Interne geneeskunde:	Namens de afdeling urologie:
Naam: ....v. Twillert..............	Naam:...............A. Claessen..............
Handtekening:	Handtekening:.......................................

ISO 9001:2000 KEMA

amc
Academisch Medisch Centrum
Universiteit van Amsterdam

Afdeling Urologie
Prof. Dr. J.J.M.C.H. de la Rosette

Drs. M. de Jongh
Dr. M.P. Laguna
Dr.med. M. Oelke
Dr.med. A. Meißner
Dr. Th.M. de Reijke
Dr.Ir. H. Wijkstra

Secretariaat Staf Urologie
Postadres: G4
Telefoon: 020 - 566 6004
Fax.: 020 - 691 9647
secr: H.C.Schaap@amc.uva.nl

Programmasecretaris ZonMw
Doelmatigheidsonderzoek, deelprogr. Implem.
Mevr. Geertje Appel
Postbus 93243
2509 AE Den Haag

28 januari 2008

Betreft: Participatie in project
(Cost)-Effectiveness of two different strategies to improve the quality of antibiotic use in patients with urinary tract infections (UTIs) in the hospital.

Projectnummer: 80-82315-98-09004

Geachte mevrouw Appels,

Hierbij verklaren wij dat wij, als deelnemend centrum, willen participeren aan bovenstaande studie. De projectleiders (Dr. S.E. Geerlings of Dr. J.M. Prins) hebben ons uitgelegd wat deelname voor ons inhoudt.

Met vriendelijke groet,
namens de afdeling Interne geneeskunde:

Dr. S.E. Geerlings

namens de afdeling urologie:

Dr. Th.M. de Reijke, uroloog

Interne Geneeskunde

ZIEKENHUIS **Rijnstate**

BEZOEKADRES
Wagnerlaan 55

POSTADRES
Postbus 9555
6800 TA Arnhem

TELEFOON 026 - 378 88 88
FAX 026 - 378 78 78

Datum 23 januari 2008

Programmasecretaris ZonMw
Afdeling: Doelmatigheidsonderzoek, deelprogramma Implementatie
Ter attentie van: Geertje Appel
postbus 93243
2509 AE DEN HAAG

Betreft: Participatie aan project (Cost)-Effectiveness of two different strategies to improve the quality of antibiotic use in patients with urinary tract infections (UTIs) in the hosptial, projectnummer: 80-82315-98-09004

Geachte mevrouw Appel,

Hierbij verklaren wij dat wij als deelnemend centrum willen participeren aan bovenstaande studie. De projectleiders (Dr. S.E. Geerlings of Dr. J.M. Prins) hebben ons uitgelegd wat deelname voor ons inhoudt.

Met vriendelijke groet,

Naam ziekenhuis: Rijnstate Ziekenhuis

Te: Arnhem

Namens de afdeling Interne geneeskunde:
Naam: EH Gisolf
Handtekening:

Namens de afdeling urologie:
Naam: P.C. Weijerman
Handtekening:

BovenIJ ziekenhuis

Programmasecretaris ZonMw
Doelmatigheidsonderzoek, deelprogramma Implementatie
Geertje Appel
Postbus 93243
2509 AE Den Haag
doelmatigheidsonderzoek@zonmw.nl

23........ Januari 2008

Betreft: Participatie aan project (Cost)-Effectiveness of two different strategies to improve the quality of antibiotic use in patients with urinary tract infections (UTIs) in the hosptial, projectnummer: 80-82315-98-09004

Geachte mevrouw Appels,

Hierbij verklaren wij dat wij als deelnemend centrum willen participeren aan bovenstaande studie. De projectleiders (Dr. S.E. Geerlings of Dr. J.M. Prins) hebben ons uitgelegd wat deelname voor ons inhoudt.

Met vriendelijke groet,

Naam ziekenhuis: Boven IJ ziekenhuis

Te: Amsterdam

Namens de afdeling Interne geneeskunde:	Namens de afdeling urologie:
Naam: M.G.W. Barnas<br>Internist<br>BovenIJ Ziekenhuis<br>Amsterdam<br>020-6346458	Naam:..
Handtekening: [signature]	Handtekening:..

167229

Bolswarderbaan 1
8601 ZK Sneek

Postbus 20 000
8600 BA Sneek

antonius ziekenhuis

Telefoon
(0515) 48 88 88

Telefax
(0515) 48 88 80

www.antonius-frl.nl

datum

uw kenmerk
ons kenmerk
doorkiesnr.
e-mail

Programmasecretaris ZonMw
Doelmatigheidsonderzoek, deelprogramma Implementatie
Geertje Appel
Postbus 93243
2509 AE Den Haag
doelmatigheidsonderzoek@zonmw.nl

...21.. Januari 2008

Betreft: Participatie aan project (Cost)-Effectiveness of two different strategies to improve the quality of antibiotic use in patients with urinary tract infections (UTIs) in the hosptial, projectnummer: 80-82315-98-09004

Geachte mevrouw Appels,

Hierbij verklaren wij dat wij als deelnemend centrum willen participeren aan bovenstaande studie. De projectleiders (Dr. S.E. Geerlings of Dr. J.M. Prins) hebben ons uitgelegd wat deelname voor ons inhoudt.

Met vriendelijke groet,

Naam ziekenhuis: Antonius ziekenhuis

Te: Sneek

Namens de afdeling Interne geneeskunde:

Naam: Dr. S.D.J.M. Niemeijer-Kanters, internist, Antonius Ziekenhuis

Handtekening: [signature]

Namens de afdeling urologie:

Naam: M. Bekker, uroloog

Handtekening: [signature]

Universitair Medisch Centrum
Utrecht

Programmasecretaris ZonMw
Doelmatigheidsonderzoek, deelprogramma Implementatie
Geertje Appel
Postbus 93243
2509 AE Den Haag
doelmatigheidsonderzoek@zonmw.nl

…25….. Januari 2008

Betreft: Participatie aan project (Cost)-Effectiveness of two different strategies to improve the quality of antibiotic use in patients with urinary tract infections (UTIs) in the hospital, projectnummer: 80-82315-98-09004

Geachte mevrouw Appels,

Hierbij verklaren wij dat wij als deelnemend centrum willen participeren aan bovenstaande studie. De projectleiders (Prof.Dr.I.M.Hoepelman,internist/infectioloog en M.T.W.T.Lock,uroloog) hebben ons uitgelegd wat deelname voor ons inhoudt.

Met vriendelijke groet,

Universitair Medisch Centrum Utrecht,PO BOX 85500,3508GA……………………………………

Te………Utrecht……………………………………

Namens de afdeling Interne geneeskunde:

Prof.Dr.I.M.Hoepelman

Handtekening: ……………………………

Prof. Dr. I. M. Hoepelman
Interne Geneeskunde & Infectieziekten
UMC Utrecht, hpnr. F.02.126
Heidelberglaan 100, 3584 CX Utrecht
tel. 31-88-7556228, fax. 31-30-2523741
I.M.Hoepelman@umcutrecht.nl

Namens de afdeling urologie:

M.T.W.T.Lock

M.T.W.T. Lock, uroloog
UMC Utrecht, C 04.236
Postbus 85500, 3508 GA Utrecht
tel. 088 - 755 8079
m.t.w.t.lock@umcutrecht.nl

Handtekening:……………………………………

urologie

De Boelelaan 1117
1081 HV Amsterdam

postbus 7057
1007 MB Amsterdam

telefoon 020 444 4444
fax 020 642 5085

www.VUmc.nl
urol@VUmc.nl

Programmasecretaris ZonMw
Doelmatigheidsonderzoek, deelprogramma Implementatie
Geertje Appel
Postbus 93243
2509 AE Den Haag
doelmatigheidsonderzoek@zonmw.nl

VU medisch centrum

datum	ons kenmerk	telefoon	fax
23 januari 2008		020 - 444 0272	020 - 444 6031
onderwerp	uw kenmerk/uw brief van	e-mail	bijlage(n):
		urol@vumc.nl	

Betreft:
Participatie aan project (Cost)-Effectiveness of two different strategies to improve the quality of antibiotic use in patients with urinary tract infections (UTIs) in the hosptial,
projectnummer: 80-82315-98-09004

Geachte mevrouw Appels,

Hierbij verklaren wij dat wij als deelnemend centrum willen participeren aan bovenstaande studie. De projectleiders (Dr. S.E. Geerlings of Dr. J.M. Prins) hebben ons uitgelegd wat deelname voor ons inhoudt.

Met vriendelijke groet,

VU medisch centrum
Amsterdam

Namens de afdeling urologie:

Prof. Dr. B.L.H. Bemelmans

Handtekening: ....

algemene / functionele urologie
prof. dr. B.L.H. Bemelmans, hoofd

oncologische urologie
dr. R.J.A. van Moorselaar

kinderurologie
mw. dr. G.M.A. Beckers
H.J.R. van der Horst

andrologische urologie
prof. dr. E.J.H. Meuleman
minimaal invasieve urologie
J.W. Mazel

research
dr. P.A.M.J. Frantzen

Programmasecretaris ZonMw
Doelmatigheidsonderzoek, deelprogramma Implementatie
Geertje Appel
Postbus 93243
2509 AE Den Haag
doelmatigheidsonderzoek@zonmw.nl

Sint Lucas Andreas Ziekenhuis

31-01 Januari 2008

Betreft: Participatie aan project (Cost)-Effectiveness of two different strategies to improve the quality of antibiotic use in patients with urinary tract infections (UTIs) in the hosptial, projectnummer: 80-82315-98-09004

Geachte mevrouw Appels,

Hierbij verklaren wij dat wij als deelnemend centrum willen participeren aan bovenstaande studie. De projectleiders (Dr. S.E. Geerlings of Dr. J.M. Prins) hebben ons uitgelegd wat deelname voor ons inhoudt.

Met vriendelijke groet,

Naam ziekenhuis: St. Lucas Andreas Ziekenhuis

Te: Amsterdam

Namens de afdeling Interne geneeskunde:

Naam: i/o dr. Veenstra
K. Lettinga

Handtekening: [signature]

Namens de afdeling urologie:

Naam: E. van Haarst

Handtekening: [signature]

St. Antonius Ziekenhuis

Het St. Antonius Ziekenhuis
vormt samen met
Mesos Medisch Centrum
de AntoniusMesosGroep

Programmasecretaris ZonMw
Doelmatigheidsonderzoek, deelprogramma Implementatie
Geertje Appel
Postbus 93243
2509 AE Den Haag
doelmatigheidsonderzoek@zonmw.nl

…….. Januari 2008

Betreft: Participatie aan project (Cost)-Effectiveness of two different strategies to improve the quality of antibiotic use in patients with urinary tract infections (UTIs) in the hosptial, projectnummer: 80-82315-98-09004

Geachte mevrouw Appels,

Hierbij verklaren wij dat wij als deelnemend centrum willen participeren aan bovenstaande studie. De projectleiders (Dr. S.E. Geerlings of Dr. J.M. Prins) hebben ons uitgelegd wat deelname voor ons inhoudt.

Met vriendelijke groet,

Naam ziekenhuis……St. Antonius Ziekenhuis……

Te……Nieuwegein……

Namens de afdeling Interne geneeskunde:	Namens de afdeling urologie:
Naam: ……M. Koene……	Naam: ……P. Vijverberg…… dr P.L.M. Vijverberg uroloog
Handtekening: ……	Handtekening: ……

Maatschap Urologie

St. Antonius Ziekenhuis

Polikliniek
Tel. (030) 609 20 05

Secretariaat
Tel. (030) 609 30 16

Urologen
R.Th. Bijleveld
A.A.G.M. Giesbers
M.G. Onaca
Dr. P.L.M. Vijverberg

Mesos Medisch Centrum

Locatie Oudenrijn
Tel. (030) 295 34 35

Locatie Overvecht
Tel. (030) 263 85 60

Urologen
K.W.H. Gisolf
Mw. E.F.H. van der Linden
A.R. Meijer

Spreekuur
Volgens afspraak

Koekoekslaan 1
Postbus 2500
3430 EM Nieuwegein

tel. (030) 609 91 11
www.antonius.net

06263

St. Antonius Ziekenhuis

Het St. Antonius Ziekenhuis
vormt samen met
Mesos Medisch Centrum
de AntoniusMesosGroep

inwendige ziekten

internisten

dr. h.s. biemond- moeniralam
dr. d.h. biesma
dr. w.j.w. bos
dr. a.b.m. geers
dr. p.chr. de jong
drs. a.j. meinders
dr. p.h.th.j. slee
dr. g. veth
dr. h.h. vincent
drs. o. de weerdt

dienstsein 81.806

maag-, darm- en leverartsen:

dr. p.a.m. van hees
drs. p.h.g.m. stadhouders
dr. m.f.j. stolk
dr. r. timmer
dr. b.l.a.m. weusten

dienstsein 81.808

reumatoloog:

dr. e.j. ter borg

secretariaat

030 - 609 20 88

telefax:

030 - 605 63 57

e-mail:

internsecr@antonius.net

spreekuur

volgens afspraak,
030 - 609 20 15 of
030 - 609 21 00

huisartsentelefoonlijn

030 - 609 20 17

Koekoekslaan 1
Postbus 2500
3430 EM Nieuwegein

tel. (030) 609 91 11
www.antonius.net

Programmasecretaris ZonMw
Doelmatigheidsonderzoek, deelprogramma
Implementatie
Geertje Appel
Postbus 93243
2509 AE Den Haag
doelmatigheidsonderzoek@zonmw.nl

…….. Januari 2008

Betreft: Participatie aan project (Cost)-Effectiveness of two different strategies to improve the quality of antibiotic use in patients with urinary tract infections (UTIs) in the hosptial,
projectnummer: 80-82315-98-09004

Geachte mevrouw Appels,

Hierbij verklaren wij dat wij als deelnemend centrum willen participeren aan bovenstaande studie. De projectleiders (Dr. S.E. Geerlings of Dr. J.M. Prins) hebben ons uitgelegd wat deelname voor ons inhoudt.

Met vriendelijke groet,

Naam ziekenhuis……ANTONIUS ZIEKENHUIS

Te……….NIEUWEGEIN……….

Namens de afdeling Interne geneeskunde: Namens de afdeling urologie:

Naam: W/W Bos internist
Naam:……dr Vijverberg: uroloog heeft separaat getekend

Handtekening: …………[signature]…………
Handtekening:……………………………………

UMC St Radboud

Universitair Medisch Centrum
Cluster Inwendige Specialismen
Algemeen Interne Geneeskunde

Huispost 463
Postbus 9101
6500 HB Nijmegen

UMC St Radboud Centraal, route 463
Geert Grooteplein 8

T (024) 361 47 63
F (024) 354 17 34

www.umcn.nl

Hoofd prof.dr. J.W.M. van der Meer
Plv. hoofd prof.dr. J.W.M. Lenders

463

Programmasecretaris ZonMw
Doelmatigheidsonderzoek, deelprogramma Implementatie
Geertje Appel
Postbus 93243
2509 AE Den Haag
doelmatigheidsonderzoek@zonmw.nl

Datum 8 februari 2008
Ons kenmerk JvdM/yg/028-08
Onderwerp Participatie aan project (Cost)-Effectiveness of two different strategies to improve the quality of antibiotic use in patients with urinary tract infections (UTIs) in the hosptial, projectnummer: 80-82315-98-09004

Geachte mevrouw Appels,

Hierbij verklaren wij dat wij als deelnemend centrum willen participeren aan bovenstaande studie. De projectleiders (Dr. S.E. Geerlings of Dr. J.M. Prins) hebben ons uitgelegd wat deelname voor ons inhoudt.

Met vriendelijke groet,

Naam ziekenhuis UMC St Radboud

Te Nijmegen

Namens de afdeling Algemeen Interne geneeskunde:

Naam: Prof. Dr. J.W.M. van der Meer

Handtekening: ....

Dienstdoend arts
T (024) 361 11 11, sein 1313
(buiten kantooruren: sein 1360)
Polikliniek
Mw. dr. L.D. Elving
Kliniek
Dr. S.J.H. Bredie
Spoedeisende hulp
Dr. ir. M. van Deuren
Onderwijs
Dr. C.T. Postma
Mw. dr. P.J.M. van Gurp

Diabetes Mellitus
Prof.dr. C.J.J. Tack
Mw. dr. L.D. Elving
Dr. F.C. Huvers
Mw. drs. B.M. Cools
Dr. G.M.M. Vervoort
Dr. B.E. de Galan
Mw. dr. P.J.M. van Gurp
Klinische farmacologie
Prof.dr. P. Smits
Dr. C. Kramers
Dr. G.A. Rongen

Infectie & afweer
Prof.dr. B.J. Kullberg
Prof.dr. J.W.M. van der Meer
Dr. P.P. Koopmans
Mw. dr. M. Keuter
Dr. A.J.A.M. van der Ven
Dr. R. van Crevel
Dr. M.G. Netea
Immunologie
Dr. Ir. M. van Deuren
Tropische Geneeskunde
Mw. dr. M. Keuter
Dr. A.J.A.M. van der Ven

Hypertensie & Vasculaire Pathologie
Prof.dr. J.W.M. Lenders
Dr. J. Deinum
Dr. C.T. Postma
Dr. S.J.H. Bredie
Dr. H.C.H. Wollersheim
Hyperlipidemie & andere Metabole Ziekten
Prof.dr. A.F.H. Stalenhoef
Prof.dr. P.M.J. Stuyt
Dr. S.J.H. Bredie
Mw. dr. J. de Graaf
Mw. dr. M.C.H. Janssen

Kamer van Koophandel - handelsregister 41055629

Kennemer Gasthuis

Programmasecretaris ZonMw
Doelmatigheidsonderzoek, deelprogramma Implementatie
Geertje Appel
Postbus 93243
2509 AE Den Haag
doelmatigheidsonderzoek@zonmw.nl

11.... februari 2008

Betreft: Participatie aan project (Cost)-Effectiveness of two different strategies to improve the quality of antibiotic use in patients with urinary tract infections (UTIs) in the hosptial, projectnummer: 80-82315-98-09004

Geachte mevrouw Appels,

Hierbij verklaren wij dat wij als deelnemend centrum willen participeren aan bovenstaande studie. De projectleiders (Dr. S.E. Geerlings of Dr. J.M. Prins) hebben ons uitgelegd wat deelname voor ons inhoudt.

Met vriendelijke groet,

Naam ziekenhuis: Kennemer Gasthuis

Te: Haarlem

Namens de afdeling Interne geneeskunde:

Naam: R. Soetekouw

Handtekening: [signature]

Namens de afdeling urologie:

Naam: ....

Handtekening: ....

1062597

Kennemer
Gasthuis

Programmasecretaris ZonMw
Doelmatigheidsonderzoek, deelprogramma Implementatie
Geertje Appel
Postbus 93243
2509 AE Den Haag
doelmatigheidsonderzoek@zonmw.nl

30 Januari 2008

Betreft: Participatie aan project (Cost)-Effectiveness of two different strategies to improve the quality of antibiotic use in patients with urinary tract infections (UTIs) in the hosptial, projectnummer: 80-82315-98-09004

Geachte mevrouw Appels,

Hierbij verklaren wij dat wij als deelnemend centrum willen participeren aan bovenstaande studie. De projectleiders (Dr. S.E. Geerlings of Dr. J.M. Prins) hebben ons uitgelegd wat deelname voor ons inhoudt.

Met vriendelijke groet,

Naam ziekenhuis: Kennemer Gasthuis

Te: Haarlem

Namens de afdeling Interne geneeskunde:	Namens de afdeling urologie:
Naam:	Naam: J.H. van der Veen, uroloog
Handtekening:	Handtekening: [signature]

LG62597

flevoziekenhuis

Programmasecretaris ZonMw
Doelmatigheidsonderzoek, deelprogramma Implementatie
Geertje Appel
Postbus 93243
2509 AE Den Haag
doelmatigheidsonderzoek@zonmw.nl

4 februari 2008

Betreft: Participatie aan project (Cost)-Effectiveness of two different strategies to improve the quality of antibiotic use in patients with urinary tract infections (UTIs) in the hosptial, projectnummer: 80-82315-98-09004

Geachte mevrouw Appels,

Hierbij verklaren wij dat wij als deelnemend centrum willen participeren aan bovenstaande studie. De projectleiders (Dr. S.E. Geerlings of Dr. J.M. Prins) hebben ons uitgelegd wat deelname voor ons inhoudt.

Met vriendelijke groet,

Flevoziekenhuis

Te Almere

Namens de afdeling Interne geneeskunde:	Namens de afdeling urologie:
Naam:	Naam: B. Meijer
Handtekening:	Handtekening: [signature]

280 400 4

Programmasecretaris ZonMw
Doelmatigheidsonderzoek, deelprogramma Implementatie
Geertje Appel
Postbus 93243
2509 AE Den Haag
doelmatigheidsonderzoek@zonmw.nl

23 Januari 2008

Betreft: Participatie aan project (Cost)-Effectiveness of two different strategies to improve the quality of antibiotic use in patients with urinary tract infections (UTIs) in the hosptial, projectnummer: 80-82315-98-09004

Geachte mevrouw Appels,

Hierbij verklaren wij dat wij als deelnemend centrum willen participeren aan bovenstaande studie. De projectleiders (Dr. S.E. Geerlings of Dr. J.M. Prins) hebben ons uitgelegd wat deelname voor ons inhoudt.

Met vriendelijke groet,

Naam ziekenhuis: Flevoziekenhuis

Te: Almere

Namens de afdeling Interne geneeskunde:	Namens de afdeling urologie:
Naam: Dr. J. Branger (per 01.05. 2008)	Naam: ....
Handtekening: [signature]	Handtekening: ....

Programmasecretaris ZonMw
Doelmatigheidsonderzoek, deelprogramma Implementatie
Geertje Appel
Postbus 93243
2509 AE Den Haag
doelmatigheidsonderzoek@zonmw.nl

21 Januari 2008

Betreft: Participatie aan project (Cost)-Effectiveness of two different strategies to improve the quality of antibiotic use in patients with urinary tract infections (UTIs) in the hosptial, projectnummer: 80-82315-98-09004

Geachte mevrouw Appels,

Hierbij verklaren wij dat wij als deelnemend centrum willen participeren aan bovenstaande studie. De projectleiders (Dr. S.E. Geerlings of Dr. J.M. Prins) hebben ons uitgelegd wat deelname voor ons inhoudt.

Met vriendelijke groet,

Naam ziekenhuis VU medisch Centrum..

Te Amsterdam...

Namens de afdeling Interne geneeskunde:

Naam: Dr. M.A. van Agtmael

Handtekening: ....

Namens de afdeling urologie:

Naam:...

Handtekening:...

Programmasecretaris ZonMw
Doelmatigheidsonderzoek, deelprogramma Implementatie
Geertje Appel
Postbus 93243
2509 AE Den Haag
doelmatigheidsonderzoek@zonmw.nl

.... Januari 2008

Betreft: Participatie aan project (Cost)-Effectiveness of two different strategies to improve the quality of antibiotic use in patients with urinary tract infections (UTIs) in the hosptial, projectnummer: 80-82315-98-09004

Geachte mevrouw Appels,

Hierbij verklaren wij dat wij als deelnemend centrum willen participeren aan bovenstaande studie. De projectleiders (Dr. S.E. Geerlings of Dr. J.M. Prins) hebben ons uitgelegd wat deelname voor ons inhoudt.

Met vriendelijke groet,

Naam ziekenhuis.... UMC St. Radboud

Te.............. Nijmegen ....

Namens de afdeling Interne geneeskunde:	Namens de afdeling urologie:
Naam:	Naam: JA Witjes
Handtekening:	Handtekening: [illegible]

**Naam en plaats ziekenhuis**	**Verantwoordelijk internist**	**Verantwoordelijk uroloog**
1. Academisch Medisch Centrum Amsterdam	Suzanne Geerlings s.e.geerlings@amc.nl	Theo de Reyke t.m.dereyke@amc.nl
2. Antoniusziekenhuis Sneek	Suzan Niemeijer sniemeijer@antonius-sneek.nl	Mariette Bekker m.bekker@antonius-sneek.nl
3. BovenIJ ziekenhuis Amsterdam	Michel Barnas M.Barnas@bovenij.nl	Ruud Vleeming r.vleeming@bovenij.nl
4. Flevoziekenhuis Almere	Judith Branger	Boaz Meijer bmeijer@flevoziekenhuis.nl
Diakonessenhuis Utrecht	*Willem Hustinx whustinx@diakhuis.nl*	Karin van Dalen kvdalen@diakhuis.nl
5. Hagaziekenhuis den Haag	Rob Valentijn r.valentijn@hagaziekenhuis.nl	Frank Froeling f.froeling@hagaziekenhui.nl
6. Kennemergasthuis Haarlem	Robin Soetekouw soetekouw@kg.nl	Ruud vd Veen jhvdveen@kg.nl
7. Medisch Centrum Alkmaar	Willem Bronsveld w.bronsveld@mca.nl	Siebe Bos s.d.bos@mca.nl
8. Onze Lieve Vrouwe Gasthuis Amsterdam	Kees Brinkman k.brinkman@olvg.nl	George van Andel g.vanandel@olvg.nl
9. Rijnstaete Arnhem	Jet Gisolf JGisolf@alysis.nl	Philip Weijerman pweijerman@alysis.nl
10. Canisius Wilhelmina ZhNijmegen	Ton Dofferhoff a.dofferhoff@cwz.nl	Herbert karthaus h.karthaus@cwz.nl
11. Rodekruisziekenhuis Beverwijk	Gitte van Twillert gvantwillert@rkz.nl	Anoesjka Claessen aclaessen@rkz.nl
12. St. Antoniusziekenhuis Nieuwegein	Willem Jan Bos w.bos@antonius.net	Peter Vijverberg p.vijverberg@antonius.net
13. St. LucasAndreas Ziekenhuis Amsterdam	Jan Veenstra j.veenstra@slaz.nl	Ernst van Haarst e.vanhaarst@slaz.nl
14. Twee Stedenziekenhuis Tilburg	Marjo van Kasteren m.vankasteren@planet.nl	Rob Davits rdavits@tsz.nl
15. Universitair Medisch Centrum Nijmegen Radboud	Jos van der Meer j.vandermeer@aig.umcn.nl	Fred Witjes F.witjes@uro.umcn.nl
16. Universitair Medisch Centrum Utrecht	Andy Hoepelman i.m.hoepelman@umcutrecht.nl	Tycho Lock m.t.w.t.lock@umcutrecht.nl
17. Vrije Universiteit Medisch Centrum Amsterdam	Michiel van Agtmael agtmael@vumc.nl	Bart Bemelmans b.bemelmans@vumc.nl
18. Ziekenhuis Amstelveen	Leslie Noach leno@zha.nl	Cobi Reisman c.reisman@planet.nl

We do not yet have letters of 3 of the particpating hospitals and will send these letters as soon as possible.

Only one internal medicine specialist (Willem Hustinx from Diakonessenhuis Utrecht) has not yet agreed to participate, but even without this hospital we have 18 participating hospitals.
