# Supplementary material for: A Cluster-Randomized Trial of Two Strategies to Improve Antibiotic Use for Patients with a Complicated Urinary Tract Infection
Source: PLoS One. 2015 Dec 4;10(12):e0142672. doi: 10.1371/journal.pone.0142672 (PMC4670093; doi:10.1371/journal.pone.0142672)
Supplement: S1 Table — (DOC) [file pone.0142672.s004.doc]

**Supporting Information**

|  | **Departments** (n) |
| --- | --- |
| **Interdisciplinary** education session(s) for other medical specialists, residents, fellows and nurses | **9**  (5 MFS + 4 CFS) |
| Education session(s) during meetings of the hospital’s antibiotic committee | **2**  (1 MFS + 1 CFS) |
| Education session(s) during hospital’s infectious diseases meetings | **2**  (1 MFS + 1 CFS) |
| Displaying poster-sized reminder letters in examination rooms at the emergency department and wards | **2**  (1 MFS + 1 CFS) |
| Changing the content of the local antibiotic guideline for complicated UTIs, concerning for example:   - recommendations for empirical therapy (in accordance with national guideline) - recommendations for treatment duration (in accordance with national guideline) - recommendations for replacement of urinary catheter after initiation of antibiotics - recommendations for catheterization to collect a urine culture in case of anuria - recommendations for tailoring and switching (iv to oral therapy) | **6**  (3 MFS + 3 CFS) |
| Changing the digital position of the local antibiotic guideline (making it more visible) to facilitate using it | **1**  (1 MFS) |
| Changing the hospital’s organizing structure:   - Urine cultures also analyzed and reported during weekends (in contrary to only during the week) - Together with a urine sediment, a urine culture is **always** collected to ensure that a culture is available if needed - Considering the implementation of an automatic stop order for antibiotic treatment (*in progress, not implemented yet at the end of the study)* | **3**  (2 MFS + 1 CFS) |

**Table 1. Additional improvement actions, performed by the MFS and CFS departments**
